# Prediction of Osteoporotic Hip Fracture Outcome: Comparative Accuracy of 27 Immune–Inflammatory–Metabolic Markers and Related Conceptual Issues

**DOI:** 10.3390/jcm13133969

**Published:** 2024-07-07

**Authors:** Alexander Fisher, Leon Fisher, Wichat Srikusalanukul

**Affiliations:** 1 Department of Geriatric Medicine, The Canberra Hospital, ACT Health, Canberra 2605, Australia; 2 Department of Orthopaedic Surgery, The Canberra Hospital, ACT Health, Canberra 2605, Australia; 3 Medical School, Australian National University, Canberra 2601, Australia; 4 Frankston Hospital, Peninsula Health, Melbourne 3199, Australia

**Keywords:** hip fracture, immuno-inflammatory–metabolic dysregulation, biomarkers, outcomes, prediction, postoperative myocardial injury, mortality

## Abstract

**Objectives**: This study, based on the concept of immuno-inflammatory–metabolic (IIM) dysregulation, investigated and compared the prognostic impact of 27 indices at admission for prediction of postoperative myocardial injury (PMI) and/or hospital death in hip fracture (HF) patients. **Methods**: In consecutive HF patient (n = 1273, mean age 82.9 ± 8.7 years, 73.5% females) demographics, medical history, laboratory parameters, and outcomes were recorded prospectively. Multiple logistic regression and receiver-operating characteristic analyses (the area under the curve, AUC) were used to establish the predictive role for each biomarker. **Results**: Among 27 IIM biomarkers, 10 indices were significantly associated with development of PMI and 16 were indicative of a fatal outcome; in the subset of patients aged >80 years with ischaemic heart disease (IHD, the highest risk group: 90.2% of all deaths), the corresponding figures were 26 and 20. In the latter group, the five strongest preoperative predictors for PMI were anaemia (AUC 0.7879), monocyte/eosinophil ratio > 13.0 (AUC 0.7814), neutrophil/lymphocyte ratio > 7.5 (AUC 0.7784), eosinophil count < 1.1 × 10^9^/L (AUC 0.7780), and neutrophil/albumin × 10 > 2.4 (AUC 0.7732); additionally, sensitivity was 83.1–75.4% and specificity was 82.1–75.0%. The highest predictors of in-hospital death were platelet/lymphocyte ratio > 280.0 (AUC 0.8390), lymphocyte/monocyte ratio < 1.1 (AUC 0.8375), albumin < 33 g/L (AUC 0.7889), red cell distribution width > 14.5% (AUC 0.7739), and anaemia (AUC 0.7604), sensitivity 88.2% and above, and specificity 85.1–79.3%. Internal validation confirmed the predictive value of the models. **Conclusions**: Comparison of 27 IIM indices in HF patients identified several simple, widely available, and inexpensive parameters highly predictive for PMI and/or in-hospital death. The applicability of IIM biomarkers to diagnose and predict risks for chronic diseases, including OP/OF, in the preclinical stages is discussed.

## 1. Introduction

Predicting postoperative complications and mortality in hip fracture (HF) patients is exceptionally complex. Most patients with HF are old, frail, have multiple comorbidities, and, consequently, a low physiological reserve.

Although a wide variety of models has been proposed for predicting HF outcomes, accurate prognosis remains among the most pressing challenges; a comprehensive review and comparison of prognostic efficacy of these models is currently lacking. The complexity and heterogeneity of HFs and the exact root causes driving adverse effects are not completely understood; a lack of a conceptional explanation of the mechanistic biological basis that underpin adverse effects is among the main reasons for suboptimal HF management.

The main factors responsible for an osteoporotic HF (as well as most chronic disorders) and its outcome include genetic predisposition and environmental, lifestyle, socioeconomic, age- and gender-related factors, all of which affect homeostasis (in this paper the terms “homeostasis” (self-regulated ability to maintain the stability of physiological processes) and “homeoresis” (constant dynamical adaptations necessary for survival) are combined under the umbrella of homeostasis) through interconnected and tightly coordinated dynamic biological mechanisms. Varying degrees of immune (innate and adaptive)*,* inflammatory, metabolic, and nutritional responses in different combinations contribute to the predisposition to HFs and their outcomes. Physiological or pathological activation of the immune–inflammatory–metabolic responses (IIMRs) have profound effects on function and survival. Dysregulation in IIMRs is a hallmark of ageing and progressive musculoskeletal deterioration, as well as diverse pathologies and life-threatening conditions in most organ systems.

Imbalances between immune, inflammatory, and biochemical processes, particularly in the ageing population (e.g., inflammageing), affect various homeostatic systems, create vicious cycles, increase the risk of multiple chronic noncommunicable diseases (including osteoporotic fracture (OF), sarcopenia, atherosclerosis, cardiovascular, neurodegenerative, renal, lung, liver, autoimmune, metabolic diseases, T2DM, etc.), and contribute to frailty and longevity. Dysregulations in metabolism are known as important determinants of the immune and inflammatory responses and vice versa (pleiotropic immune-metabolic interplay) [1,2,3,4,5,6,7,8,9,10,11,12,13,14,15,16,17,18,19,20,21]. Assuming that poor HF outcome is linked to and reflects the failure of interconnected homeostatic mechanisms, an evolutionary highly conserved complex and dynamic process, it appears logical to identify abnormal IIMRs as prognostic factors to correct the reversable one.

**Figure 1:**
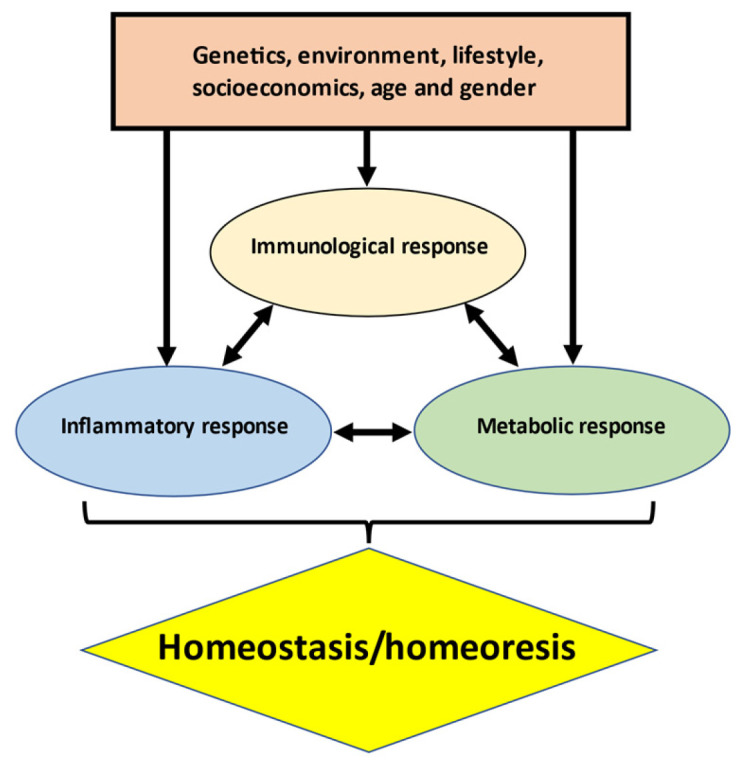
Schematic overview of main determinants of immune–inflammatory–metabolic (IIM) homeostasis in health and disease. The diagram illustrates complex dynamic and toughly interconnected immune, inflammatory, and biochemical processes—the three main hallmarks of homeostasis. These evolutionary integrated processes (feedback loops) are regulated and influenced by numerous genetic, environmental, lifestyle, socioeconomic, age- and gender-related factors via myriads of signalling pathways. Analysis of individual IIM status provides a unified understanding of ageing, pathology, and progression of most chronic diseases and indicates the potential diagnostic, prognostic, preventive, and therapeutic targets.

Figure 1 depicts schematically the physiologically inseparable links between three main pillars of homeostatic regulation which involve various pathways integrating diverse metabolic, immune, and inflammatory functions. Immuno-inflammatory–metabolic (IIM) imbalances caused by environmental stresses, poor lifestyle choices, and/or physiological conditions lead to tissue damage and constitute the “common soil” [2] of most chronic diseases, including osteoporosis (OP) and OF. From this perspective, a person’s IIM state provides a close representation of an individual’s overall health status and reflects what has been encoded by the genome and modified by environmental, lifestyle, and disease-related factors.

Although in recent years numerous novel biochemical, immunological and inflammation biomarkers have gained significant attention as predictors of clinical outcomes in different settings, the most reliable candidates to prognosticate HF outcome at hospital admission remain unknown.

To our knowledge, no study summarised the proposed outcome models in HF patients, and there is no consensus on which one is the best for predicting the risk of a poor outcome. Among numerous outcome predictors in the published literature, we have chosen 27 simple blood indices with established regulatory functions, focussing on reversable/modifiable parameters, and compared the prognostic value of each of these tests at admission in HF patients. Driven by elementary logic, we attempted to find pragmatic, convenient, easy to apply, and pathogenically important indices that can provide effective pre-surgery predictive information and, hence, may constitute good targets for preventive and therapeutic interventions. To characterise the IIMRs, we analysed a large group of peripheral blood cell (red blood cells, neutrophils, lymphocytes, monocytes, eosinophils, and platelets) counts, red blood cell distribution widths (RDWs), haemoglobin, serum albumin, alanine aminotransferase (ALT), and gamma-glutamyl transferase (GGT) levels, and we calculated 18 different ratios, including the neutrophil-to-lymphocyte ratio (NLR), platelet-to-lymphocyte ratio (PLR), monocyte-to-lymphocyte ratio (MLR), systemic immune–inflammation index (SII), systemic inflammation response index (SIRI), etc. (Table 1). These simple and affordable indicators of IIM imbalance(s) demonstrated prognostic significance in many different diseases and are increasingly used as biomarkers of poor survival, particularly, in malignancy. However, about one-third of these indices have never been applied to predict HF outcomes. We used two distinct outcome variables corresponding with the main research questions: occurrence of postoperative myocardial injury (PMI) and the in-hospital mortality.

The aim of this study was twofold. First, to evaluate and compare the predictive performance, accuracy, and reliability of each of the 27 potential predictive biomarkers (including those not covered by any previous study), considering the patient’s age and history of ischaemic heart disease (IHD), and to provide recommendations for the best tests that would indicate at admission a high risk of PMI and/or lethal outcome. Second, to present an overview of the potential utility of the IIM biomarkers in early identifying patients at a risk of OP/OF (and related disorders) and individualising preventive strategies.

## 2. Materials and Methods

### 2.1. Patients

In this observational single-centre study, we analysed prospectively collected data on a cohort of 1273 consecutive patients (older than 60 years) admitted with a low-trauma non-pathological HF (cervical or trochanteric) to the Department of Orthopaedic Surgery of Canberra Hospital (a university-affiliated tertiary care centre) between 2010 and 2019 who underwent operative fracture treatment. The present study extends our previous work; detailed descriptions of this cohort, inclusion, and exclusion criteria have been published [22]. In brief, patients with high- or medium-energy fractures, multiple fractures, polytrauma, pathological (malignant tumour) or subtrochanteric fractures were excluded; a low-energy mechanism was defined as a fall from no greater than standing height. The mean age of patients was 82.9 ± 8.7 [SD] years, 73.5% were women, and 50.5% had a cervical fracture. All patients followed a similar postoperative protocol, with mobilisation out of bed on day one and a urinary catheter being taken out on day two.

The validation cohort (n = 582, mean age 81.9 ± 9.13 years, 71.0% women, 52.9% with a cervical fracture) had a similar to the main cohort profile of chronic comorbid diseases, admission laboratory characteristics and outcomes.

### 2.2. Data Collection

Data on socio-demographic (including pre-fracture residential status, use of walking aid) characteristics, lifestyle factors (smoking, alcohol use), clinical (14 chronic comorbidities, medications used) and laboratory parameters at admission (within 12–24 h of arrival), type of surgery, and postoperative hospital outcomes were prospectively recorded and analysed.

Comorbidities included ischaemic heart disease (IHD), prior myocardial infarction (MI), hypertension (HT), cerebrovascular accident (CVA), transient ischaemic attack (TIA), type 2 diabetes mellitus (T2DM), atrial fibrillation (AF), chronic kidney disease (CKD), anaemia, chronic obstructive airway disease (COPD), dementia, Parkinson’s disease (PD), and rheumatic and malignant (without bone metastasis) diseases. The diagnoses of IHD, hypertension, T2DM, and all other chronic diseases were based on current guidelines and documentation in the previous hospital and general practitioners’ medical case records.

### 2.3. Laboratory Measurements

The routine laboratory tests included full blood count, serum electrolytes, creatinine, urea nitrogen, C-reactive protein (CRP), albumin and liver function tests, cardiac troponin I (cTnI), 25(OH) vitamin D [25(OH)D], intact PTH, thyroid stimulatory hormone (TSH), free thyroxine (T_4_), vitamin B_12_, folic acid, iron, ferritin, and transferrin; analyses were performed by standard laboratory methods using auto-analysers. Serum calcium concentrations were corrected for serum albumin, and the glomerular filtration rate was estimated (eGFR). Serum cardiac troponin I (cTnI) levels were assessed pre- and within 24 h postoperatively and then after if elevated and/or clinically indicated. All patients with an elevated cTnI level of >20 ng/L or greater (“abnormal” laboratory threshold) were assessed for ischaemic features (ischaemic symptoms and 12-lead electrocardiogram). Chronic kidney disease (CKD) was defined as an estimated glomerular filtration rate (eGFR) < 60 mL/min/1.73 m^2^.

The studied IIM parameters and cut-offs used are listed in Table 1. The optimal cut-off values for most IIM parameters have not yet been established; reported reference data vary substantially across different studies reflecting patient variability, differences in demographics, race/ethnicities, underlying disease, comorbidities, complications, etc.

In this study, the absolute cut-offs for single haematological indices have been defined according the existing consensus definitions. The robustness of cut-offs for some ratios was validated in our prior studies ([23,24,25,26,27,28]); for other cut-offs, median values have been used. Our cut-offs for composite parameters are in accordance with a number of values reported in the literature. For example, some studies found optimal cut-off values for NLR of 6.14 [29,30] or 8.16 [31], for PLR > 204.4 [32], and a ratio of 4.41 [33]–5.87 [34] for Plt/Alb; our Alb/RDW ratio is also close to that used by other researchers [35,36,37,38].

### 2.4. Outcome Measures

These included (1) PMI defined by cTnI rise (if, on days 1–5 post-surgery, at least one cTnI measurement was >20 ng/L with or without associated ischemic symptoms); (2) a high inflammatory response assessed by marked elevation of CRP (>100 mg/L after the 3rd postoperative day); (3) length of hospital stay (LOS); (4) all-cause in-hospital mortality. Currently, there is no consensus on recommendations regarding the threshold levels of cTnI elevations for the definition of perioperative myocardial infarction even in patients undergoing cardiac surgery (the proposed cutoffs range from >10 times to ≥70 times the upper reference limit) [39]. Because most of our HF patients were asymptomatic and not candidates for (and did not have) a coronary angiogram, in this study, postoperative AMI was conditionally defined by cTnI ≥ 500 ng/L (25 times above the upper limit of reference levels) accompanied by obvious ECG signs (Q-waves, ST-segment changes, T-wave inversion) indicative of myocardial ischemia and supportive transthoracic echocardiographic signs (i.e., regional wall motion abnormalities, exclusion of non-coronary artery disease causes of ST-segment elevation). In accordance with current guidelines, all patients with PMI have been treated with dual anti-platelets (usually aspirin and clopidogrel and balancing the bleeding risk), b-blockers (routinely metoprolol and avoiding bradycardia /hypotension), and lipid lowering drugs (mainly statins); other medications (diuretics, calcium channel blockers, renin–angiotensin–aldosterone system inhibitors, and angiotensin-receptor antagonists have also been considered if clinically indicated) and special attention to fluid balance was given.

### 2.5. Statistical Analyses

Data analyses were carried out using Stata software version 16 (Stata Corp., College Station, TX, USA). Continuous variables (if normally distributed) were reported as numbers (means ± SD) and categorical variables as percentages. Comparisons between groups were performed using analysis of variance and a Student’s *t*-test for continuous variables and an χ^−2^ test (Yates corrected) for categorical variables. Univariate and multivariate (both linear and logistic) regression analyses were used to determine the odds ratio (OR) and 95% confidence intervals (CIs) for associations between an outcome (dependent variable) and different clinical and laboratory variables; all potential confounding variables with statistical significance ≤ 0.15 on univariate analyses were included in the final multivariate analyses. We presented the results of unadjusted, minimally adjusted, and fully adjusted analyses concurrently. Associations between IIM markers were assessed using a Pearson correlation coefficient with a Bonferroni adjustment. A receiver operating characteristic (ROC) curve analysis (the area under the ROC curve, AUC) was used to investigate the discriminatory power of preoperative indices to predict postoperative events. An AUC between 0.7 and 0.8 was considered acceptable, between 0.8 and 0.9 excellent, and higher than 0.9 outstanding. Sensitivity, specificity, accuracy, positive predictive value (PPV), negative predictive value (NPV), positive likelihood ratio (LP+), negative likelihood ratio (LP−), and number of patients needed to be examined for correct prediction (NNP) [40,41] were calculated to assess the discriminatory performance of the tests. NNP (1/[PPV + NPV − 1]) is considered a better descriptor of diagnostic/prognostic tests in populations with different prevalences of the disease [40,41]; low NNP values are desirable. The predictive performance of the models was further assessed using goodness-of-fit statistics for calibration by a Hosmer–Lemeshow test. All tests were two-tailed; statistical significance was set at *p* values < 0.05.

## 3. Results

### 3.1. Baseline Characteristics and Outcomes

The sociodemographic data, comorbidities, and outcomes in the analysed cohort of HF patients were presented in detail in our previous paper [22]. Shortly, of 1273 consecutive patients who underwent HF surgery, 361 (28.4%) had previously been diagnosed with IHD and 99 subjects (7.8% of the total cohort, 27.4% among IHD patients) had a history of acute myocardial infarction (AMI). Several differences were detected between IHD and non-IHD groups. HF patients with IHD compared to the non-IHD were significantly older (+2.7 years on average); had a higher prevalence of hypertension, CKD, chronic obstructive pulmonary disease (COPD), cerebrovascular accident (CVA), type 2 diabetes mellitus (T2DM), and Parkinson’s disease; more often used walking aids; and were less likely to be female and alcohol over-users. The percentage of active and ex-smokers, permanent residential care facilities (PRCF) residents, and patients with different fracture types (cervical or trochanteric), dementia, anaemia, and TIA did not differ in these two groups. Patients with IHD, compared to the non-IHD persons, as would be expected, more often developed PMI (58.6% vs. 37.7%, *p* < 0.001), AMI (11.7% vs. 4.8%, *p* < 0.001) and had a high inflammatory response (CRP > 100 mg/L in 84.2% vs. 79.7%, *p* = 0.037) and prolonged hospital stay (LOS > 20 days in 25.8% vs. 20.4%, *p* = 0.024).

PMI occurred in 555 (43.6%) patients, including 58.6% in the IHD group and 62.1% among patients with a history of AMI. Compared to the rest of the cohort, patients with PMI, not surprisingly, more frequently had a history of IHD (37.7% vs. 20.6%, *p* < 0.001), AMI (11.1% vs. 5.2%, *p* = 001), hypertension (60.2% vs. 51.4%, *p* = 0.001), TIA (12.6% vs. 8.3, *p* = 0.009), anaemia (46.0% vs. 38.4%, *p* = 0.005), and dementia (38.5% vs. 26.2%, *p* < 0.001), were older (+5.3 years), were more often >80 years of age (85.2% vs. 60.4%, *p* < 0.001), males (28.9% vs. 24.6%, *p* = 0.054), and PRCF residents (38.7% vs. 28.4%, *p* < 001), but were less likely to be alcohol over-users (1.9% vs. 5.4%), current smokers (4.1% vs. 6.5%) or suffering from Parkinson’s disease (3.8% vs. 5.9%). Additionally, history of stroke, COPD, T2DM, smoking (ex) and use of walking aids were not associated with PMI. A total of 6.7% patients experienced postoperative AMI, including 11.7% with previously known IHD and 4.8% without IHD. PMI was observed most often in the first 1–3 days after surgery (when patients were receiving analgesic medications that can mask ischaemic symptoms) and was asymptomatic in 97.8% of these patients. PMI was symptomatic only in 15 individuals, including 9 with postoperative AMI; in most patients, the myocardial injury would probably have gone undetected without routine cTnI measurements. PMI was associated with high inflammatory responses (CRP > 150 mg/L in 69.2% vs. 55.1%, *p* < 0.001) and LOS > 10 days (61.4% vs. 54.9%, *p* = 0.013). The total all-cause in-hospital mortality was 4.8%, in patients without IHD −7%, with IHD −7.5% (*p* = 0.005), and in those with previous AMI −11.8%. PMI increased risk of a lethal outcome 5-fold (OR 5.0, 95% CI 2.70–9.41, *p* < 0.001). The mortality rate in patients who developed PMI was 8.8% (vs. 1.9% in the non-PMI subjects, *p* < 0.001), in the group with known IHD −12.9%, and among individuals with a history of AMI −15.8%. IHD patients with a fatal outcome compared to survivors were older (88.6 ± 5.34 vs. 84.6 ± 7.23, *p* = 0.006), all but one > 80 years of age (96.3% vs. 75.1%, *p* = 0.006) and more often had CKD (70.4% vs. 44.0%, *p* = 0.007), while all other examined sociodemographic (including male sex prevalence: 9.1% vs. 6.8%, *p* = 0.285) and comorbid characteristics did not show statistical differences between the groups.

To summarise, in HF patients, the presence of IHD (after controlling for age, gender, HF type, preoperative residence, mobility status, comorbidities) increased the risk of a fatal outcome by 2-fold (OR 2.1, 95% CI 1.24–3.51, *p* = 0.005), of developing PMI by 2.3-fold (OR 2.3, 95% CI 1.81–3.01, *p* < 0.001) and a postoperative AMI by 2.4-fold. Among all patients who died, 56 (87.5%) were aged > 80 years, 48 (78.7%) experienced PMI, and 27 (42.3%) had a history of IHD. Furthermore, in subjects aged > 80 years with a history of IHD, the risk of PMI was 8.3 times higher (OR 8.3, 95% CI 5.58–12.36, *p* < 0.001) and risk of a lethal outcome was 7.4 time higher (OR 7.4, 95% CI 2.55–21.51, *p* < 0.001) compared to HF patients without such characteristics. Moreover, both IHD and PMI were also associated with high postoperative inflammatory responses and prolonged hospital stays.

These findings are in line with previous studies and confirm the utility of advanced age, history of IHD, and developing PMI for elucidating the prognosis and identifying the highest risk groups. However, it should be recognised that most of aged HF patients, even with known IHD and/or PMI (including new AMI), survive suggesting the need of more precise prediction tools, especially for individuals with the above-mentioned characteristics. In other words, these results indicate that history of IHD and advanced age have a significant but limited value in predicting outcomes in older HF patients; these clinical characteristics are not sufficiently reliable and valid, and tests with a higher accuracy/sensitivity are required. Therefore, we further evaluated the possible role of different haematological indices of IIM disbalance at admission as risk factors and predictors of PMI and/or in-hospital death, focusing on high-risk patients with poor prognosis.

### 3.2. Association between the IIM Indices at Admission and IHD

Table 2 summarises the baseline patient haematological indices, stratified by the presence of IHD. Analysis showed that, in patients with IHD, compared to the non-IHD group, 10 parameters differed significantly. Namely, among patients with IHD (who were older and more likely males), there was a higher proportion of subjects with a lower number of RBC and eosinophils, an elevated (above the normal range) number of monocytes, and RDW, and, consequently, dysbalanced ratios—Neutr/Eos, Mon/Eos, Hb/RDW, RDW/Plt × 100, Alb/RDW, and Plt/Alb.

### 3.3. IIM Indices and Postoperative Outcomes (Univariate Analysis)

Baseline haematological characteristics in patients with regard to outcomes are listed in Table 3. Univariate analysis showed that many haematological parameters at admission, along with older age, were associated with poor outcomes—PMI and/or in-hospital death. PMI was associated with 12 indices, a fatal outcome—with 17 laboratory characteristicsand 10 biomarkers were indicative for both outcomes. For PMI, the following indices showed significant ORs (in 9 of them the OR was above 1.40): LMR < 1.1 (OR 1.78), RDW > 14.5% (OR 1.57), SIRI > 5.1 (OR 1.52), PLR > 280 (OR 1.49), Hb/RDW < 8.9 (OR 1.48), monocyte count > 1.0 × 10^9^/L (OR 1.46), NLR > 7.5 (OR 1.46), SII > 1620 (OR 1.41), Alb/RDW > 2.6 (OR 1.40), anaemia (OR 1.36), lymphocyte count <1.2 × 10^9^/L (OR 1.36), Neutr/Alb > 2.4 (OR 1.30). For in-hospital death, statistically significant ORs demonstrated the following admission biomarkers (nine of which had an OR between 2.7 and 2.0): Alb/RDW > 2.6 (OR 2.70), Neutr/Eos > 156.3 (OR 2.49), Hb/RDW < 8.9 (OR 2.49), SII > 1620.0 (OR 2.43), RDW > 14.5% (OR 2.35), LMR < 1.1 (OR 2.31), PLR > 280 (OR 2.29), SIRI > 5.1 (OR 2.13), eosinophil count < 0.5 × 10^9^/L (OR 2.00), NLR > 7.5 (OR 1.81), Hb/Alb < 4.6 (OR 1.81), Plt/Alb > 5.9 (OR 1.80), GGT/Lymp > 25.4 (OR 1.70), Neutr/Alb > 2.5 × 10 (OR 1.69), Mon/Eos > 13.0 (OR 1.69), anaemia (OR 1.68). Notably, two biomarkers (lymphocyte count < 1.2 × 10^9^/L and monocyte count > 1.0 × 10^9^/L) were suggestive only for developing PMI, whereas seven other indices were indicative only for a fatal outcome, despite PMI and hospital death being significantly interrelated. These observations, taken together, suggest that simple haematological tests at admission reflect different pathophysiological factors and mechanisms responsible for these outcomes and may be clinically valuable for predicting PMI and/or hospital mortality.

### 3.4. Comparison of IIM Indices in Patients with Postoperative Myocardial Injury with and without Pre-Fracture Diagnosed IHD

To further verify the predictive role of admission IIM characteristics and their relationship with IHD, we performed a subgroup analysis in patients who developed PMI, comparing the clinical profile and the IIM biomarkers in subjects with and without known IHD pre-fractures (Table 4). Patients with PMI and previously undiagnosed IHD, compared to those with known IHD, more often were female (74.1% vs. 66.2%, *p* = 0.050) and more often had been diagnosed with dementia (41.6% vs. 33.3%, *p* = 0.056), but significantly fewer had CKD (37.9% vs. 54.7%, *p* < 0.001) and COPD (13.9 vs. 20.9%, *p* < 0.034); among subjects with IHD who developed PMI, 23.4% had had an AMI in the past. In the percentages of patients with an abnormal eosinophil count, the RDW, RDW/Plt, Hb/RDW, Neutr/Eos and Mon/Eos ratios were higher among the IHD group, whereas 21 other biomarkers did not show significant differences between the groups; the non-IHD group exhibited a tendency for higher incidence of Plt/Alb > 5.9 (50.5% vs. 41.9%, *p* < 0.057), and these patients were more likely to progress to a lethal outcome (67.4% vs. 45.3%, *p* = 0.004).

In subjects with PMI, the incidence of high postoperative response (CRP > 100 mg/L or >150 mg/L), prolonged LOS (>10 or >20 days), and mortality rate were not influenced by a pre-fracture diagnosis of IHD. In other words, the main adverse hospital outcomes in HF patients who developed PMI should be attributed to the underlying pathophysiological factors, including IIM dysregulation (most components of which, as shown by the studied biomarkers, are common for patients with and without IHD), but not to IHD per se.

Taken together, this analysis revealed that many haematological parameters at admission are potential indicators of developing PMI (regardless of IHD presence) and may be particularly helpful in females and patients with dementia in the absence of an IHD history.

### 3.5. Relationships between IIM Indices (Pearson’s Correlation)

Although the studied peripheral blood IIM indices reflect different aspects of the complex IIM system, many of them are significantly interrelated. Indeed, a matrix of pairwise Pearson coefficients of correlation (with Bonferroni adjustment) produced for all 27 biomarkers confirmed numerous significant associations between the haematological indices. For example, to name a few, LMR < 1.1 was positively correlated with neutrophil count (r = 0.2331, *p* < 0.001), lymphocyte count < 1.1 (r = 0.4089, *p* < 0.001), eosinophil count < 0.5 (r = 0.2487, *p* < 0.001), monocyte count > 1.0 (r = 0.2924, *p* < 0.001), Alb/Lymph < 25.4 (*p* = 0.4584, *p* < 0.001), Mon/Eos > 13 (r = 0.3002, *p* < 0.001), SII >1620.0 (r = 0.3912, *p* < 0.001), Neutr/Alb × 10 > 2.4 (r = 0.2627, *p* < 0.001), RDW >14.5% (r = 0.0737, *p* = 0.009), Alb/RDW < 2.6 (r = 0.0996, *p* = 0.004), and albumin < 33 g/L (r = 0.0675, *p* < 0.016); the LMR < 1.1 was significantly negatively correlated with GGT/Lymph >25.4 (*p* = −0.2600, *p* < 0.001), Neutr/Eos > 156.3 (*p* = −0.2684, *p* < 0.001), ALT/Lymph < 25.4 (*p* = −0.2808, *p* < 0.001), and Hb/Alb < 4.6 (*p* = −0.0860. *p* = 0.002), but LMR < 1.1 was not correlated with high platelet count, Plt/ALT, Neutr/Mon, and Plt/Alb ratios. Similarly, Neutr/Eos > 156.3 was correlated with eosinophil (r = 0.8529, *p* < 0.001), neutrophil (r = 0.3635, *p* < 0.001), and lymphocyte (r = 0.2923, *p* < 0.001) counts, as well as NLR >7.5 (r = 0.4535, *p* < 0.001), LMR < 1.1 (r = 0.2684, *p* < 0.001), SII >1620.0 (r = 0.3983, *p* < 0.001), GGT/Lymph > 25.4 (r = 0.1113, *p* = 0.001), Alb × Lymph < 25.4 (r = 0.2549, *p* < 0.001), ALT/Lymph < 14.6 (r = 0.2818, *p* < 0.001), Mon/Eos >13.0 (r = 0.8567, *p* < 0.001), Neutr/Alb × 10 > 2.4 (r = 0.3181, *p* < 0.001), and Neutr/Mon > 12.1 (r = 0.3087, *p* < 0.001) ratios, but not with high platelet count, low albumin, or abnormal Plt/Alb, Alb/ RDW, and Hb/Alb ratios.

Age > 80 years correlated with RBC < 4.30 × 10^12^/L (r = 0.0752, *p* = 0.007), anaemia (r = 0.1059, *p* = 0.002), abnormal (according to abovementioned cut-offs) RDW (r = 0.1181, *p* < 0.001), NLR (r = 0.0774, *p* = 0.006), PLR (r = 0.0600, *p* = 0.032), LMR (r = 0.0727, *p* = 0.009), SII (r = 0.0716, *p* < 0.011), lymphocyte count (r = 0.1000, *p* = 0.001), Plt/ALT (r = 0.1098, *p* = 0.001), RDW/Alb (r = 0.1315, *p* < 0.001), Alb × Lymph (r = −0.1280, *p* < 0.001), as well as with eGFR (r = 0.1968, *p* < 0.001), PTH (r = 0.0924, *p* = 0.001) and vitamin D < 50 nmol/L (r = −0.0825, *p* = 0.0035). Advanced age did not correlate with elevated neutrophil, monocyte or platelet count, or with low eosinophil count, low albumin level, Mon/Eos, Neutr/Alb, Plt/Alb, Hb/Alb, GGT/Lymph, Neutr/Eos, and ALT/Lymph ratios.

Theoretically and practically, the indices of dysregulated IIM homeostasis, although interrelated, differ from each other, and each reflects specific disturbances; it is important to acknowledge these relationships when interpreting clinical findings. The extent to which different biomarkers are present in a patient reflects the complexity and severity of the IIM dysregulation(s).

### 3.6. Independent Predictors of Poor Hospital Outcome

Multivariate regression, which included all laboratory variables significantly associated with PMI as well as all variables with *p* <0.150 on univariate analysis, age, and gender, showed that, in the total HF cohort, independent predictors for PMI were as follows: LMR < 1.1 (OR 1.39, 95% CI 1.01–1.92, *p* < 0.0.047), RDW > 14.5% (OR 1.34, 95% CI 1.05–1.72, *p* < 0.019), age > 80 years (OR 3.80, 95% CI 2.83–5.09, *p* < 0.001) and male gender (OR 1.48, 95% CI 1.12–1.95, *p* < 0.006). When clinical characteristics/comorbidities were added to the model, the significance of RDW > 14.5% diminished and became nonsignificant (OR 1.23, 95% CI 1.01–1.58, *p* < 0.108). The final model incorporated LMR < 1.1 (OR 1.58, 95% CI 1.20–2.09, *p* = 0.0.001), age > 80 years (OR 3.23, 95% CI 2.38–4.38, *p* < 0.001), IHD (OR 2.04, 95% CI 1.56–2.67, *p* < 0.001), CKD (OR 1.72, 95% CI 1.33–2.22, *p* < 0.001), dementia (OR 1.39, 95% CI 1.07–1.81, *p* = 0.013), and male gender (OR 1.42, 95% CI 1.06–1.88, *p* = 0.017). This model, containing six characteristics, collectively explained 10.3% (R^2^) of the variance in the PMI outcome and yielded an AUC of 0.7263 (95% CI 0.6629–0.7897); of note, low LMR on admission independently increased the risk of developing PMI by 58%.

The same methodological approach as for the development of PMI was used in the multivariate logistic regression for predicting hospital death. In the model which included all laboratory characteristics, as well as IHD, age and sex, the independent predictors of a fatal outcome were as follows: Neutr/Eos > 156.3 (OR 2.28, 95% CI 1.22–4.25, *p* < 0.008), Alb/RDW < 2.6 (OR 1.92, 95% CI 10.96–3.65, *p* < 0.045), age > 80 years (OR 4.25, 95% CI 1.66–10.89, *p* = 0.003) and IHD (OR 1.86, 95% CI 1.08–3.21, *p* < 0.025). The final model after adjustment for all clinical characteristics enclosed four admission variables as independent predictors (Neutr/Eos > 156.3 (OR 2.91, 95% CI 1.64–5.15, *p* < 0.001), Alb/RDW < 2.6 (OR 2.50, 95% CI 1.39–4.49, *p* < 0.002), age > 80 years (OR 3.98, 95% CI 1.55–10.21, *p* = 0.004), and CKD (OR 2.52, 95% CI 1.47–4.31, *p* = 0.001)), whereas IHD lost significance (OR 1.63, 95% CI 0.94–3.62, *p* = 0.082); this model explained 13.6% (R^2^) of the variance of the lethal outcome and yielded an AUC of 0.7544 (95% CI 0.6952–0.8136); notably, an on-admission high Neutr/Eos ratio and low Alb/RDW ratio independently indicated an increased risk of hospital mortality by 191% and 150%, respectively.

To sum up, independent determinants of poor/fatal outcome in HF patients include IIM dysregulation along with advanced age, IHD, CKD, and dementia; different components of IIM dysregulation are integrated in and expressed by combined biomarkers such as LMR < 1.1, Alb/RDW < 2.6, and Neutr/Eos > 156.3. The pathophysiological heterogeneity of underlying mechanisms indicates the importance of subgroup analyses considering clinical characteristics.

### 3.7. Prognostic Value of On-Admission IIM Characteristics

Different IIM indices showed clinical usefulness as prognosticators for poor hospital outcome, especially when HF patients were stratified into groups representing IHD and aged ≥ 80 years (Table 5). In the total HF cohort, age ≥ 80 years near quadrupled (OR 3.84) and history of IHD doubled (OR 2.09) the PMI risk; multivariate analyses showed that 7 of 27 studied parameters at admission indicated PMI, as follows: LMR < 1.1 (OR 1.56), PLR > 280.0 (OR 1.44), SII > 1650.0 (OR 1.56), SIRI > 5.1 (OR 1.42), NLR > 7.5 (OR 1.40), eosinophil count < 0.5 × 10^9^/L (OR 1.38) and Mon/Eos > 13.0 (OR 1.38). In IHD patients, the presence of any one of these seven biomarkers further significantly increased the risk of developing PMI (the ORs ranged between 2.50 and 3.87 (being above 3.30 for five indices)); moreover, in subjects with IHD, 10 other admission biomarkers also indicated a PMI risk (OR ranged between 2.32 and 3.22, being 2.50 and above for eight biomarkers). In the aged > 80 years IHD patients, PMI risk was 8.3-times higher than in the rest of the cohort, and the presence of indices of IIM dysregulation further increased this risk by 1.5–2-fold; additionally, 17 biomarkers demonstrated an OR of 16.36–8.49 (13 indices had an OR ≥ 11.10).

Compared to the rest of the cohort, risk of in-hospital death was 2.1 times higher in subjects with IHD, 4.9 times higher in aged patients, and 7.4 times higher in patients >80 years of age with a history of IHD (Table 5). More than half (15 of 27) of on-admission IIM parameters were significantly associated with a lethal outcome; the highest OR demonstrated the following 13 indices: Alb/RDW < 2.6 (OR 2.71), Neutr/Eos > 156.3 (OR 2.49), eosinophil count < 0.5 × 10^9^/L (OR 2.44), SII > 1620.0 (OR 2.33), PLR > 280.0 (OR 2.16), SIRI > 5.1 (OR 2.13), LMR < 1.1 (OR 2.03), Mon/Eos > 13.0 (OR 2.02), Hb/RDW < 8.8 (OR 1.92), RDW > 14.5% (OR 1.86), ALT/Lymph < 14.6 (OR 1.75), NLR > 7.5 (OR 1.72), and Neutr/Alb × 10 > 2.4 (OR 1.69). In the IHD group, the presence of specific haematological biomarkers further increased risk of mortality up to 2.5-fold: PLR > 280.0 (OR 5.58), LMR < 1.1 (OR 5.19), eosinophil count < 0.5 × 10^9^/L (OR 4.76), SIRI > 5.1 (OR 3.95), Plt/Alb ratio > 5.9 (OR 3.95), Mon/Eos > 13.0 (OR 3.93). The risk of in-hospital death in IHD patients aged >80 years (compared to total IHD group) was 2–6 times higher if they had PLR > 280.0 (OR 29.21), LMR < 1.1 (OR 28.67), Neutr/Alb × 10 >2.4 (OR 23.06), GGT/Lymph > 25.4 (OR 20.53), lymphocyte count < 1.1 × 10^9^/L (OR 18.6), or eosinophil count < 0.5 × 10^9^/L (OR 13.96).

It is worth noting that most preoperative haematological variables and their ratios were in the “normal range” (median values as cut-offs), or only mildly differed from them, and are commonly considered as non-diagnostic and non-prognostic. Moreover, a number of haematological parameters prognostically not significant when the total HF cohort was analysed (e.g., anaemia, low lymphocyte count, elevated neutrophil, monocyte, or platelet counts, hypoalbuminaemia, as well as particular ratios—RDW/Plt, Plt/ALT, Neutr/Mon, and Alb/Lymph) demonstrated usefulness when clinical characteristics (history of IHD, advanced age) were considered.

Our data suggest that, in the total HF cohort, the presence of at least one of one biomarkers on arrival increased the risk of PMI by 33–56%, whereas 11 indices increased the risk of a fatal outcome by 70–149%; among subjects with IHD, 26 biomarkers (except elevated platelet count) increased PMI risk by 105–287% and 23 biomarkers indicated a 123–458% higher risk of a fatal outcome, while in IHD patients aged >80 years, the corresponding numbers were approximately 2–6 times higher. These findings emphasise again that, to identify HF patients on admission with the highest risk of a poor outcome, each laboratory prognostic index should be interpreted in conjunction with patient’s clinical characteristics, in particular age and history of IHD.

### 3.8. Predicting Performance of On-Admission IIM Characteristics for Hospital Outcome

Next, we assessed the ability and accuracy of different models to predict poor outcomes in HF patients focusing on the highest risk groups (Table 6). Each model included one haematological parameter (single or composite) at admission and two clinical characteristics (history of IHD and advanced age). A receiver operating characteristic analysis was performed to evaluate the predictive power of each model. Of note, although most evaluated biomarkers in the aged (>80 years) or/and IHD groups demonstrated a significant prognostic value for developing PMI and/or hospital mortality (high ORs), not all of them had a reasonable predictive performance (Table 5 and Table 6).

The highest AUC for predicting a lethal outcome showed models with the following on-admission haematological indices: PLR >280.0 (AUC 0.8390), LMR < 1.1 (AUC 0.8375), or albumin < 33 g/L (AUC 0.7889), RDW > 14.5% (AUC 0.7739), anaemia (AUC 0.7604), eosinophil count < 0.5 × 10^9^/L (AUC 0.7540), or Neutr/Alb > 2.4 (AUC 0.7545); five more models also had considerable incremental value in predicting patients at risk of hospital death with an AUC above 0.7120. The performance parameters (sensitivity, specificity, accuracy, as well as PPV, NPV, LR+, LR−) of different biomarkers, as would be expected, varied broadly. Six tests had a good sensitivity of 90% and above (90.9% to 94.1%), and nine other tests had a sensitivity of 80–89.5%. However, only five tests demonstrated a predictive specificity of 79.3–84.6% (monocytes > 1.0 × 10^9^/L, platelets > 400 × 10^9^/L, albumin < 33 g/L, LMR < 1.1, and PLR > 280.0), whereas the majority of biomarkers had a specificity only slightly above 50% (52.1–66.6%). Accordingly, the positive predictive values (PPV) of the tests were quite low (ranging from 2.9% to 20.8%) but the negative predictive values (NPV) were very good (96.9–99.3%), meaning that survivors had been identified correctly. The prediction validity of the studied models was also assessed by the number of patients with a given condition(s) who needed to be examined in order to correctly detect/predict one person with a certain outcome (number needed to predict, NNP). The NNP, a fatal outcome in HF patients, based only on the presence of IHD was 26.3, the NNP based only on age >80 years was 20.4, and a combination of both characteristics was 12.5. The NNP decreased dramatically when the following IIM parameters at admission were considered: PLR > 280.0 (NNP = 5.03), LMR < 1.1 (NNP = 5.35), eosinophil count < 0.5 × 10^9^/L (NNP = 6.49), or albumin < 33 g/L (NNP = 6.76); for 10 other indices, the NNP ranged between 7.58 and 10.53.

**Figure 2:**
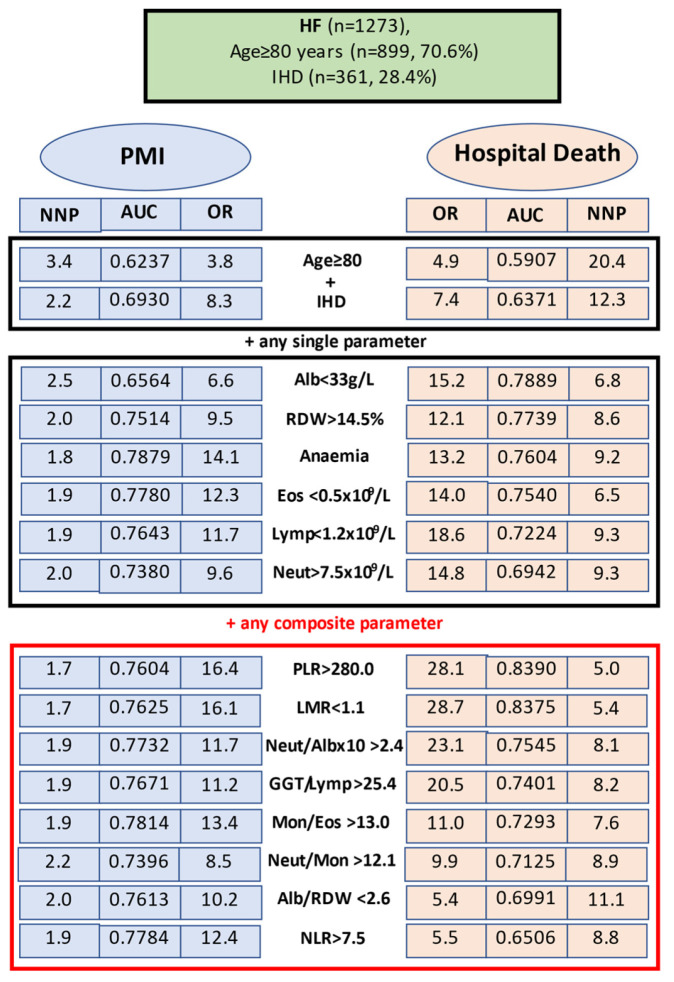
Simplified summary of selected prognostic haematological indices at admission and their performance for prediction postoperative myocardial injury (PMI) and/or in-hospital death (in order of the AUCs) in patients with hip fracture aged ≥ 80 years. The performance of single parameters (absolute counts) and combined models (based on ratios) shown. Each model includes three variables: age > 80 years, IHD, and one haematologic index. AUC ≥ 0.750 (considered acceptable) are highlighted.

With respect to PMI, a good discriminative performance with values for an AUC above 0.7600 displayed models with anaemia (AUC 0.7879), Mon/Eos > 13.0 (AUC 0.7814), NLR > 7.5 (AUC 0.7784), eosinophil count < 0.5 × 10^9^/L (AUC 0.7780), Neutr/Alb × 10 > 2.4 (AUC 0.7732), GGT/Lymph > 25.4 (AUC 0.7671), lymphocyte count < 1.2 × 10^9^/L (AUC 0.7643), LMR < 1.1 (AUC 0.7625), and PLR > 280.0 (AUC 0.7604); three more tests had an AUC above 0.7380. Six indices demonstrated sensitivity above 80% (neutrophil count < 7.5 × 10^9^/L, lymphocyte count < 1.2 × 10^9^/L, Mon/Eos > 13.0, GGT/Lymp > 25.4, RDW/Plt × 100 > 6.6 and Neutr/Mon > 12.1), and eight other tests demonstrated a sensitivity between 75% and 80%. Three tests (PLR > 280.0, LMR < 1.1 and anaemia) exhibited the highest specificity (82.1–91.4%), accuracy (79.7–82.1%), and PPV (71.1–75.0); models with 21 other biomarkers showed an NPV of 83% and above. Notably, values for the likelihood (LR+) of PMI to be predicted by these biomarkers were high (range 7.036–1.862), suggesting balance in favour of the right conclusion over misdiagnosis. The NNP of PMI based only on IHD history was 4.8, only on advanced age was 3.4, and on both characteristics it was 2.2. In combined models, with one of 10 haematological parameters added, the NNP decreased below 2.0, and the lowest NNPs (1.7–1.8) were achieved with PLR > 280.0, LMR < 1.1 and anaemia (Table 6, Figure 2).

The top 14 models for prediction PMI and/or hospital death in aged IHD patients are shown in Figure 2. The strongest predictors for development of PMI among the single haematological variables were anaemia (AUC 0.7879), low eosinophil count (AUC 0.7780), and low lymphocyte count (AUC 0.7643), and among the composite indices it was elevated Mon/Eos ratio (AUC 0.7814), the NLR (AUC 0.7784), and the Neutr/Alb ratio (AUC 0.7732). The dominant features for predicting a lethal outcome on admission were hypoalbuminaemia (AUC 0.7889), high RDW (AUC 0.7739), and anaemia (AUC 0.7604) among single indices, and high PLR (AUC 0.8390), low LMR (AUC 0.8375), and elevated Neutr/Alb ratio (AUC 0.7545) among the composite indices. In AUC analyses for the prediction of both PMI or in-hospital death, the composite indices showed, in general, a better performance than the single ones; the exceptions to this were anaemia and low eosinophil count, which obtained the highest AUCs for predicting PMI. There was, however, a substantial heterogeneity in the baseline predictive features between models reflecting the differences (despite the shared pathogenic pathways) in the dysregulated IIM homeostasis underlying perioperative (patho-) physiological changes responsible for adverse outcomes.

The tests which occupy the first 14 places in terms of the weight (the greatest AUCs) of the preoperative prediction for PMI and/or fatal outcome in the aged IHD patients (Figure 2) demonstrate similarities, confirming commonalities as well as differences in the risk factors and pathophysiological mechanisms. A number of indices have comparable overall accuracy for predicting both outcomes, whereas some tests demonstrate superior performance for predicting in-hospital death and others have superior performance for predicting PMI. As an example, NLR > 7.5 was the second strongest predictor for PMI (AUC 0.7784), but only a weak indicator of a fatal outcome (AUC 0.6506); inappropriate PLR and LMR were the strongest predictors for hospital death but were not among the best indicators for PMI. A few more examples are as follows: among the single indices, anaemia and low eosinophil count showed higher AUCs for predicting PMI than mortality, while elevated RDW demonstrated the opposite; among the composite indices, altered Mon/Eos and Neutr/Alb ratios had higher AUCs for predicting PMI than hospital death. However, two parameters—PLR > 280 and LMR < 1.1—were able to differentiate between patients with and without poor outcomes (both developing PMI and hospital death) with equal 79.7–82.2% accuracy (Table 6).

In all, in aged patients with IHD, the following models demonstrated a reasonable predictive accuracy (AUC exceeding 0.7700): five characteristics showed the highest AUC (ranged between 0.7879 and 0.7732) for predicting PMI—anaemia, Mon/Eos > 13.0, NLR, Eos < 0.5 × 10^9^/L and Neutr/Alb × 10 > 2.4, whereas the four best predictors of a fatal outcome were PLR > 280.0, LMR < 1.1, albumin < 33 g/L, and RDW > 14.5 (AUC range 0.8390–0.7739). These observations put the indices reflecting dysregulated IIM homeostasis at the centre as predictors of poor outcomes, challenging the notion that IHD and/or advanced age are enough to trigger PMI and/or cause death.

Notably, in the total cohort of HF patients, the incidence of the above-mentioned IIM biomarkers for predicting poor outcomes was also high. Namely, among 555 patients who developed PMI, only 23 (4.1%) did not have any of the five most informative haematological indices, whereas 443 (79.8%) subjects had two or more, 378 (68.1%) had ≥3, and 205 (36.9%) had >5 of the predictive characteristics. On the other hand, among 718 patients without PMI, 80 (11.1%) patients had at least one of five biomarkers. In other words, five IIM parameters on-admission identified PMI risk in most cases (532 among 555 actually observed, 95.8%), but false predictions may occur in 11.1% of patients when the indices are applied to the total cohort without considering IHD and age.

Of 61 HF patents who died in the hospital, 60 (98.4%) subjects had at least one of the five above-mentioned IIM indices at admission. Considering these five predictors, 55 (90.2%) of 61 deceased subjects exhibited two or more indices, 48 (78.7%) had ≥3, and 32 (52.5%) had ≥5 of the predictive biomarkers. Obviously, the chosen IIM tests on-admission may help to identify subjects at risk of a fatal outcome in the total cohort of HF patients: 60 fatalities could be expected, which is consistent with the actual observation (n = 61). Among 1212 survivors, at least one of five IIM biomarkers were found in 152 (12.2%) patients.

To conclude, the presented data clearly indicate that, in patients with HF, preoperative dysregulations of IIM homeostasis are common and appear as persistent processes causing adverse events. Selected IIM parameters are useful for predicting poor outcomes, and the accuracy of described models improves significantly when on-admission haematological indices are integrated with clinical characteristics.

### 3.9. Internal Validation

In the validation cohort, sociodemographic and clinical parameters, including the proportion of elderly patients (>80 years, 72.1%) and subjects with IHD (28.3%), were not significantly different from that in the derivation group; additionally, PMI was observed in 44.1%, and the all-cause mortality rate was 4.1%, indicating that the cohorts were well balanced in terms of their baseline characteristics. The fourteen best-performing indices/models in the derivation cohort (listed in Figure 2) were evaluated. The on-admission IIM indices in both cohorts produced, in general, similar (almost identical for some models) prognostic and predictive values. For example, in the validation cohort, risk for developing PMI in IHD patients aged >80 years with PLR > 280.0 was 14.4 times higher than in patients without such signs, the AUC was 0.7618, sensitivity 77.1%, specificity 81.0%, and the NNP was 1.8. In subjects with LMR < 1.1, the corresponding figures were OR 15.9, AUC 0.7611, 54.8%, 93.1%, and 1.9; in subjects with a low eosinophil count, the corresponding figures were OR 11.8, AUC 0.7560, 90.2%, 54.6%, 2.1. Additionally, in cases of Mon/Eos > 13.0, the corresponding figures were OR 9.9, AUC 0.8667, 65.1%, 84.1%, NNP 2.1. Similarly, the IIM characteristics demonstrated a reasonable high predictive value and acceptable calibration (Hosmer–Lemeshow statistic, all *p* > 0.010) for a fatal outcome. As an example, the ORs for the four abovementioned indicators ranged between 29.1 and 14.0 in the main cohort and between 31.1 and 11.0 in the validation cohort, whereas the AUC ranged between 0.8390 and 0.7293 in the main cohort and between 0.8477 and 0.7043 in the validation cohort.

Altogether, internal validation confirmed the prognostic usefulness and good discriminative performance of models based on haematological indices (on hospital arrival) to predict PMI and/or in-hospital death HF patients.

### 3.10. Practical Considerations/Application

Here we address practical issues relevant in prediction, prevention and treatment of adverse clinical outcomes in HF patients (1) and discuss the potential role of IIM biomarkers for identifying patients at risk before any visible clinical symptoms and signs of OP/OF, falls and/or related chronic disorder(s) occur (2).

#### 3.10.1. IIM Biomarkers in Assessing and Managing Short-Term Outcomes

Currently early identification of HF patients who are at high risk of PMI and/or mortality remains an unmet clinical need. Our study shows evidence that presence of an abnormal IIM characteristic at arrival should raise an alertness and suspicion of a potentially poor outcome.

Deregulation(s) in IIM homeostasis drives adverse outcomes, and this complex association is reflected by multiple biomarkers. The presented findings demonstrate that a relatively small number of simple indices on admission (chosen from the long and growing list of tests based on their predictive value) are informative for prognosis, patient stratification, and prioritising treatment intervention; there is no need to measure all overlapping modalities reflecting the myriad pathways that regulate IIM. As mentioned, at least one of five most informative/predictive haematological indices was found in 95.8% of all patients who developed PMI and in 98.4% of all subjects with a fatal outcome compared to 11.1% and 12.2% among patients without PMI and survivors, respectively. However, because of the heterogenicity of HFs’ underlying aetiology and pathophysiology, a single biomarker may not capture the breadth of the complex network involved in and responsible for adverse outcomes in different HF patients. The IIM characteristics contributing to adverse outcomes reflect different (albeit interlinked) pathophysiological mechanisms and, therefore, may be complementary for prediction decisions in HFs. Each laboratory parameter should be interpreted cautiously in conjunction with clinical data, considering its sensitivity and accuracy to avoid misclassification (both over- and underdiagnosis).

Tests with high negative predictive value (NPV), especially when the prevalence of an adverse event is low, should be applied for the exclusion of poor outcomes; conversely, tests with a modest positive predictive value (PPV) may result in overdiagnosis. Understandable indices with high sensitivity help to identify patients with a high probability of poor outcomes, while tests with high specificity may indicate that a poor outcome is unlikely. The discriminative capability of the preoperative IIM characteristics improves significantly when they are analysed in combination with known clinical factors (e.g., advanced age, history of IHD or both). For example, in HF patients aged >80 years, compared to younger individuals, the risk of developing PMI or hospital death was 3.8 and 4.9 times higher, respectively; in aged subjects with a history of IHD, the corresponding figures were 8.3 and 7.4 (compared to patients without these characteristics), and if these two clinical features were accompanied with on-admission low lymphocyte count (<1.2 × 10^9^/L), the corresponding figures were 11.7 and 18.6, while, in cases of anaemia, the risks were 14.1 and 13.2 times higher, respectively. Similarly, the risks of PMI or/and death were times higher in the aged IHD patients with eosinophil count < 0.5 × 10^9^/L (ORs 12.3 and 14.0, respectively), RDW > 14% (ORs 9.5 and 12.1, respectively), PLR > 280.0 (ORs 16.4 and 29.1, respectively), or LMR < 1.1 (ORs 16.1 and 28.7, respectively)—to mention a few predictive parameters at admission (Figure 2). Clearly, IIM characteristics interpreted in combination with clinical criteria better determine an individual’s prognosis as well as the need and eligibility for specific therapy. On a practical level, for predicting HF outcome at admission, IIM indices should be added to the standard clinical evaluation starting with analysis of routine single blood biomarkers, and if the prognosis remains unclear/doubtful, consideration of the patient’s age and comorbidities should be used to assess the composite biomarkers (Figure 2). Clinicians can expect that, out of every 100 HF patients aged >80 years with IHD, a high risk (>83%) of developing PMI will demonstrate individuals with an on-admission high neutrophil count (85.4%), low lymphocyte count (84.3%), or high Mon/Eos ratio > 13.0 (83.1%); a high incidence (≥90.0%) of a lethal outcome should be expected among patients with a high neutrophil count (95.0%), low lymphocyte count (95.0%), high Neutr/Alb ratio (94.7%), or low eosinophil count (90.0%).

As an illustration, in aged IHD patients with HF, a quick and valid predictive result at admission regarding a possible fatal outcome relies on the following three simple variables: age, history of IHD, and anaemia, as well as low absolute count of eosinophils or lymphocytes, elevated RDW percentage, and (if still in doubt) abnormal ratios of PLR or LMR. The predictive values of high PLR and low LMR far exceeded that of altered platelet, lymphocyte, or monocyte counts. Data on a comprehensive family of indices to be used for risk adjustment purposes and accurate prediction of specific outcomes in HF patients, summarised in Figure 2, may help clinicians to choose the optimal prognostic test in the highest risk group. Analysis based on 2–3 biomarkers (and viewed in the context of clinical findings) is preferable to avoid both an unrecognised poor outcome and false alarms.

Remarkably, PMI occurred in 211 (58.4%) of 361 patients with known IHD, whereas in 344 (62.0%) of 555 patients who developed PMI, pre-fracture IHD had not been diagnosed. The all-cause in-hospital mortality rate among IHD patients was 7.5%, and it was 8.3% in the PMI group, indicating the limited prognostic value of pure clinical factors. In contrast to the accepted dogma, the incidence of most altered IIM biomarkers among patients with and without IHD did not differ significantly, suggesting the importance of IIM imbalance for predicting, preventing and managing both PMI and lethal outcome, events which are strongly associated but not inseparable.

Although most risk factors for poorer outcomes are not preventable (advanced age, multi-comorbidity, frailty, etc.), higher risk patients are more likely to benefit from specific therapeutic interventions. Evaluation of the IIM status represents a substantial opportunity to stratify risks and apply personalised, tailored patient-specific therapies. The potentially preventable and/or reversable factors include correction of anaemia, hypoalbuminaemia, malnutrition, low grade inflammation, disturbances in vitamin D, K, and mineral status, etc.; appropriate pharmacotherapies can be employed perioperatively.

#### 3.10.2. Role of IIM Biomarkers for Evaluation and Management of Health Status

Systemic IIM dysregulations are not only independent prognostic factors of poor outcomes but have a significant role in the development and progression of numerous diseases, particularly ones which are age-related and associated with OP/OFs.

In the context of rapid population ageing, the increasing burden of chronic health disorders (including OP/OFs), difficulties in risk assessment, predicting and preventing bone loss and falls, and the limited success of modern preventive therapies, there is a critical need to identify people at risk early to tailor individualised treatments.

Chronic conditions are, however, continuingly first diagnosed at advanced/irreversible stages when targeted therapy is less (or not) effective.

Given that OP is a disease continuum, altered IIM status should be considered a risk factor/biological predictor for developing chronic disorders over a lifetime, including OP/OF. Early identification of individuals with IIM dysregulation(s) may be particularly useful in patients stratified as a low-risk group (no previous fragility fractures, BMD T-score ≥ −1.0, FRAX-calculated 10-yaer HF risk < 3% and 10-year risk of major osteoporotic fractures < 20%). IIM parameters and their combinations might shed light on the processes that contribute to and drive disease progression, providing a further understanding of what particular characteristics need to be primarily focused on in each individual patient. The treatments may include lifestyle modification (physical activity, smoking cessation, alcohol moderation) and appropriate pharmacotherapies for specific IIM disturbances and comorbidities (CVD, CKD, T2DM, COPD, etc.). However, in the realm of public health and clinical practice, the IIM status, in comparison to traditional clinical risk factors, is not given adequate attention and rarely addressed. The approach proposed here is focused on targeting the root causes of chronic diseases (prior symptoms/signs of tissue/organ damage occur), which may substantially prevent OP/OF, as well as related chronic disorders, and extend disease-free health spans. We suggest that emerging data on the essential role of IIM dysregulations have yet to be translated in practical diagnostic, preventive and treatment strategies. Even a modest abnormality in IIM markers should facilitate discussion and review patient’s lifestyle and diet, correction of metabolic and mineral disorders, malnutrition, low-grade inflammation, etc.; musculoskeletal health (OP/OF risk) assessment and further investigation(s), if not previously done, need to be considered. Routine evaluation by primary care providers and all medical specialists of IIM parameters, particularly in middle aged and elderly persons with high risks of chronic noncommunicable diseases, may be an attractive and valuable strategy (in spite of low specificity of these indices).

Currently there is no “silver bullet” for preventing and treating OP/OFs, a heterogenous condition with complex involvement in IIM dysregulations and multiple organ systems. To target and reduce dysregulations in IIM, homeostasis appears to be a logical and effective step forward in breaking feedback loops between IIM dysregulation and multiorgan extra-skeletal pathologies linked with OP/OF, especially in earlier stages (before most risk factors have accumulated). As almost all patients with OP/OF demonstrate signs of low-grade inflammation, a condition associated with poorer short- and long-term prognosis, it appears that anti-inflammatory interventions could be beneficial in the fight against OP/OFs and related extra-skeletal disorders. Anti-inflammatory and immune-modulating mechanisms and normalisation of metabolic disturbances underlie, at least partially, the well-recognised positive effects of lifestyle modifications (physical exercise, healthy diet, smoking cessation, etc.) [42,43,44], as well as the use of traditional bone-related nutrients, including vitamins D, C, K, and E, calcium, magnesium, micronutrients (iron, zinc and selenium), probiotics, and prebiotics [9,45,46,47,48,49,50,51,52].

Moreover, recent studies have demonstrated the effectiveness of anti-inflammatory/disease-modifying drugs, in particular colchicine, canakinumab, via-2291, anakinra, hydroxychloroquine, and methotrexate in treatment of low-grade inflammation in cardiovascular disease, T2DM and other OP-related disorders, by reducing the plasma levels of pro-inflammatory cytokines (e.g., IL-6 and CRP) and improving lymphocyte, platelet and endothelial cells functions [4,43,53,54,55,56,57,58,59,60,61,62]. Among recommended drugs with the potential ability to counteract inflammatory cytokines are also metformin, aspirin, ibuprofen, rapamycin, sartuins, and statins [19]. Anti-inflammatory drugs, however, should be used with caution, and their limitations and unwanted side effects (e.g., increased infections due to depressed host defence mechanisms, or gastrointestinal upset/bleeding) should be considered. Colchicine, in tissue culture at low concentrations, inhibited selectively bone-like cell mineralisation (without affecting cell proliferation) [63]; in rats prolonged treatment with colchicine reduced bone strength and influenced fracture healing negatively [64]. Additionally, concomitant use of colchicine and drugs inhibiting cytochrome P450 3A4 and P-glycoprotein may cause potentially life-threatening drug–drug interactions (rhabdomyolysis/myopathy, myelosuppression/agranulocytosis, cardiac arrhythmias) [65,66,67,68]. Traditional non-steroidal anti-inflammatory drugs (NSAID) and selective cyclooxygenase-2 (COX-2) enzyme inhibitors, medications widely used postoperatively in orthopaedic patients for the treatment of pain and inflammation, may also cause adverse gastrointestinal, renal, and cardiovascular effects. The possible inhibitory effects of NSAID (especially the COX-2 inhibitors) on the bone healing process have been reported [69,70,71,72,73,74,75,76]; but most recent studies found that the nonunion risk was small [77,78] or negligible [79], site-, sex-, dose- and duration-dependent, and significant only in patients who received NSAIDs for >3–4 weeks [80,81]. According to available data, anti-inflammatory drugs could be beneficial in preventing cardiovascular events (known to be associated with OP/OF), but the potential risks, although rare, have to be put into perspective.

Other therapies with immunomodulatory and anti-inflammatory effects and protective roles in musculoskeletal and related disorders, such as vitamin D [14,82,83,84,85,86,87,88,89,90,91,92,93,94] and vitamin K [95,96,97,98,99,100], also deserve special attention.

A shift in the existing management paradigm focused mainly/solely on anti-resorptive treatment to a model which addresses specific IIM deregulations may be fruitful in both analysing the risks and improving the outcomes. An approach which implies biomarkers of IIM status (in contrast to the current routine strategy where treatment decisions are based mainly on BMD, resulting in delayed diagnosis and “one-size-fits-all” therapy), may help to individualise and optimise prognosis, introduce treatment early, and decrease the OP/OF burden.

Clearly, not every patient who exhibits markers of IIM dysregulation may develop OP/OF and/or any other chronic disease, but existing data suggest that a substantial proportion of subjects in whom IIM changes are detected will progress to chronic illnesses. It is quite understandable that the dilemma between overdiagnosis and overtreatment IIM dysregulations, on the one hand, and the risk of delayed intervention(s) contributing to the occurrence of chronic diseases and/or adverse events, on the other, should be considered with every individual patient. Identifying individuals in need of attention and finding the “right” IIM target(s) in each patient may be difficult. Single indices may not adequately capture varying pathways (due to heterogeneous underlying aetiologies) and predict outcomes in all patients; therefore, a multimodal approach (evaluating at least 2–3 biomarkers of IIM homeostasis) is preferable to minimise risks. Whether such a strategy would provide an advantage and reduce OP and fracture rates needs to be further investigated.

To conclude, immunometabolic dysregulation and low-grade inflammation determine the complex processes that underlie ageing, most age-related chronic diseases, and long- and short-term outcomes. Haematologic biomarkers of IIM status, aside from clinical features, provide significant diagnostic and prognostic information for early identification and disease severity stratification, reflecting specific (and interconnected) risks in different patients. Although the causes and mechanisms of IIM deregulation and tissue/organ dysfunction are still elusive, emerging evidence suggests that addressing IIM homeostasis may provide novel preventive and therapeutic strategies to improve management for many non-infectious chronic disorders associated with OP/OFs.

## 4. Discussion

Although IIM deregulation is nearly universally associated with most human diseases, it has not been fully translated into clinical prediction models or applied to preventive strategies in the field of OP/OF. The term dysregulated IIM status is used here as an umbrella term for a host of conditions that could be caused by different mechanisms but are integrated with each other and create vicious cycles. Each of the IIM components should be considered as a factor affecting various systems in the body, and, when dysregulated, they may trigger and amplify numerous pathophysiological processes and increase the risk of multiple chronic diseases, including OP/OFs.

In this paper, we assessed and compared the prognostic information and predictive performance of 27 blood-based IIM biomarkers in HF patients at admission and illuminated the potential clinical utility of IIM parameters for identification of individuals at risk of chronic disorders, including OP/OF, in the preclinical stage.

### 4.1. IIM Parameters at Admission as Predictors for Outcomes in HF Patients

Objective stratification of HF patients, prediction of postoperative complications and mortality and optimisation of management is a complex and challenging topic of crucial importance. Multiple models proposed for predicting HF outcomes (partially reviewed previously [22]) varied hugely in methodology (some require calculations, scoring systems and computer use, differ in statistical assessments of the performance, etc.), source of data, and number and availability of analysed variables, and they are largely focused on sociodemographic and clinical variables in different combinations but rarely involve haematological characteristics; due to these limitations, the predictive value of existing models is still debatable. Only a few studies have been conducted on the development and prognosis of PMI. Currently, preoperative cardiovascular risk assessment is based on clinical factors (demographics, presence of cardiac disease, and non-cardiovascular comorbidities) [101], and the pivotal involvement of IIM dysregulation(s) in this complication has not been evaluated systematically.

Our analysis revealed that, among 27 studied blood-based IIM biomarkers (only half of which had previously been applied to the prognostication of HF outcomes), in the total HF cohort, 10 indices were significantly associated with the development of PMI and 16 parameters were indicative of a fatal outcome; in the subset of patients diagnosed with IHD, the corresponding figures were 26 and 21, and, among the IHD patients aged >80 years, the corresponding figures were 26 and 20. Ten out of 27 haematological parameters at admission predicted both PMI and hospital death, but with some differences (Table 5 and Table 6). In the aged patients with a history of IHD (the highest risk group), the five strongest predictors of PMI were preoperative anaemia (AUC 0.7879), Mon/Eos ratio > 13.0 (AUC 0.7814), NLR > 7.5 (AUC 0.7784), low eosinophil count (AUC 0.7780), and Neutr/Alb × 10 > 2.4 (AUC 0.7732); the sensitivity of these models ranged between 83.1% and 75.4% and the specificity ranged between 82.1% and 75.0%. The highest risk for in-hospital death exhibited subjects with the following five on-admission indices: PLR > 280.0 (AUC 0.8390), LMR < 1.1 (AUC 0.8375), albumin < 33 g/L (AUC 0.7889), and RDW > 14.5% (AUC 0.7739), and anaemia (AUC 0.7604). Four of these models (except low albumin) had a sensitivity of 88.2% and above, and the specificity of the three first models was 85.1–79.3% (Table 6; Figure 2); all abovementioned models showed adequate discrimination and good fit in calibration.

In the total HF cohort, independent predictors (after adjusting for clinical covariates and relevant risk factors) for developing PMI were LMR < 1.1, age > 80 years, IHD, CKD, dementia, and male gender, while independent predictors of all-cause mortality were Neutr/Eos ratio > 156.3, Alb/RDW ratio < 2.6, age > 80 years, and CKD. These data indicate that, in HF patients, most of the IIM biomarkers on admission are not independent risk factors for PMI or lethal outcome but reflect the co-participating pathways, the overlapping and interrelated fundamental immune, inflammatory, endocrine, and metabolic mechanisms of IIM dysregulation(s) across most organ systems contributing to development and progression of many different diseases (including OP/OF), and, consequently, adverse HF outcome.

The overlap of prognostic value of different biomarkers indicates their common routes; on the other hand, the heterogeneity and complexity of OP/OFs means that use of prognostic biomarkers in combination (e.g., ratios of promising indices) and, specifically, with identification of clinical characteristics is necessary to optimise sensitivity and specificity. There is no single characteristic that can capture the complexity of factors responsible for poor outcome in all HF patients. Our findings suggest that a combination of three on-admission parameters—one IIM biomarker, advanced age, and history of IHD—can predict PMI and/or hospital death sufficiently. These models compare favourably with previously published research.

The prediction models for HF outcomes or developing OP/OFs reported in the literature were based on multiple variables (between five and 29), often included intra- and postoperative characteristics, and showed inconsistencies. A recent systematic review of prediction models for OF (68 studies describing 70 models) found that AUC ranged from 0.60 to 0.91. In most models (84.3%), the AUC was under 0.80, and only two models achieved AUC > 0.90 [102]; models for prediction OP in older adults had an AUC of 0.849 (seven variables examined) [103] and 0.850 (15 variables) [104]. The AUC for in-hospital death (five variables) was 0.731 [105], for 30-day mortality (40 studies included)—0.621–0.860 [106,107,108,109], for 90-day mortality (12 variables)—0.67 [110], for 6-month mortality (14 metabolites)—0.68 [111], and for 1-year mortality, following HF, the AUC ranged between 0.79 (10 variables, accuracy of 81%, sensitivity of 34% and specificity of 98%) [112]—0.797 (seven variables) [113]—0.758 (10 variables) [114] and 0.717 (seven variables) [115]. The prediction performance of newest machine learning-based models was similar: the AUC for mortality prediction (39 studies) was 0.84 [116], for HF prediction (10 factors)—0.78 (sensitivity 75% and specificity 78%) [117]; the highest AUC value for the risk of in-hospital mortality among critically ill patients with HF was 0.797 (6 parameters) [118].

Today’s dominance of multivariable architectures of most predictive models is questionable; such approaches underestimate essential physiological principles and the biological evidence that all immunological, inflammatory, and metabolic responses are integrated.

Our approach (which combined one haematological biomarker and two clinical characteristics) demonstrates the following significant advantages in predicting HF outcome: it is rapid, simple, easy-to-use, accessible to any physician, and outperforms most previously published models. Numerous clinical and laboratory factors are associated with poor outcomes, but they, as shown in our and other studies, are not independent of each other and, when combined, may add only a relatively small proportion to the predictive information on HF outcomes. This can be explained by the fact that each component of IIMR is correlated to many others and, therefore, not surprisingly, a plethora of different IIM parameters demonstrate significant prediction power. This assumption resembles George Cuvier’s principle of correlation of parts (the basic concept to comparative anatomy and palaeontology), which states that all organs in an animal’s body are deeply interdependent and, thus, the function and structure of each organ allow for us to reconstruct fossils and make predictions.

In the total Australian population in 2020, only 2.47% were aged >80 years; however, this age group made up 70.6% of the HF cohort and accounted for 85.2% among subjects who developed PMI and 91.8% among the fatalities. The risk of HF in subjects aged >80 years, compared to those younger than 80 years, was 28.2 times higher; HF patients of this age were nearly six times more likely to experience PMI and about 11 times more likely to die. These observations are in line with many recent reports [119,120,121,122,123,124,125].

Our data on the prognostic value of most (but not all) individual IIM biomarkers for predicting PMI and/or hospital death in HF patients agree with previously reported research (Table 7). The association of abnormal preoperative single blood indices with weakened immune systems, poor overall health, postoperative morbidity, and mortality has been demonstrated in countless studies in different settings. In general, the adverse postoperative outcomes clearly reflect the effects of baseline and comorbid diseases, while individual indices per se may show only a weak predictive value [126,127]. It is worth noting the complexity of IIM responses, particularly the interactions between neutrophilia, platelet and monocyte activation, suppression of lymphocytes and eosinophils, over-production of inflammatory cytokines, etc. [128,129].

In recent years, measurement of eosinophils, cells with a variety of complex immunomodulatory functions [312,313,314], has attracted substantial attention in chronic diseases (other than atopic conditions) but has not been assessed in HF patients. These studies provided contradictory results; for instance, conflicting evidence on the correlation between eosinophil count and CVD/IHD risk and outcomes have been reported, as follows: Eosinopenia was linked to acute myocardial infarction (AMI), heart failure and death in clinical studies [175,177] and preclinical models [176], and a high eosinophil count was shown to be a protective factor against coronary artery stenosis [178] and 6-month to 1-year mortality, but it was associated with long-term mortality [183,315]; other researchers, however, reported positive associations between eosinophil counts and functions with IHD risk [316] and poor CVD outcomes [146,317,318,319]. Differences in studied populations (sociodemographic and lifestyle factors, prevalence of comorbid conditions, medication used) and methodology may explain these differing publications [188].

Comparable human studies on prognostic performance of blood IIM biomarkers are scarce and the variable selection, cut-offs, and covariate-adjustment vary widely; this hampers an objective comparison between the different IIM indices and the conclusions regarding their prediction values, even in the same disease range, significantly. For example, in HF patients, all-cause in-hospital mortality was reported to be best predicted by increased RDW levels [194] as well as by an increased Neutr/Alb ratio or NLR, but not by PLR [300]; SII was found to be a stronger predictor of poor outcomes than that of PLR and NLR in patients with acute coronary syndromes [277], whereas other researchers showed that NLR, PLR, and SII have similar predictive values in NSTEMI patients [226]. A consensus on which biomarker performs better in specific diseases is still lacking.

The natural, and often irreversible, age-related changes in IIM status play, undoubtedly, a significant role in functional and morphological decline and contribute to serious complications and death [1,3,11,17,320,321]. Abnormal IIM indices in HF patients can not only aid to guide prognostic decision-making but should lead to peri-operative optimisation of modifiable risk factors (as discussed above).

### 4.2. Usefulness of Parameters of IIM Homeostasis for Early Screening, Risk Stratification, Prevention and Optimal Management of Osteoporosis and Fractures

To optimise the strategy of OP/OF management, it is of seminal importance to identify and early (at the preclinical stage) recognise the risks for alterations in musculoskeletal and related systems. Because IIM dysregulation(s) are causal and central to most human diseases, comprehensive analyses of its components hold value for understanding and predicting (with precision) the potential risks in each individual. The tripartite complex multilevel dynamic network involving the immune, inflammatory, and metabolic functions determines key mechanisms of homeostasis in health and disease. The development of OP/OFs are complex, multifactorial processes in which IIM dysfunctions have pathophysiological, therapeutic, and prognostic roles. Abnormal IIM components through numerous interconnected mechanisms trigger directly and indirectly loss of bone and muscle mass and functions (as well as affect the whole spectrum of chronic disorders associated with OP/OF); the premorbid alterations precede and underlie overt OP/OF. However, the biomarkers of IIM homeostasis have not been systematically evaluated and have not yet been used to generate evidence-based concepts of co-occurrence and the interdependence of pathological disorders in preclinical stages. Prognostication of the potential risks due to IIM dysregulations, especially among middle aged and older adults (in whom most age-related changes are often irreversible and the chronic conditions are incurable), and early implementation of specific interventions and preventive treatments to counteract changes which may contribute to functional and morphological decline, complications, and death is still an unmet goal. It is important to emphasise the utility of IIM status when screening patients for OP/OF risk and vice versa; individuals with IIM dysregulation(s) have an increased risk of different chronic disorders associated with OP/OF. Elucidating IIM status in asymptomatic persons and, in case of deviations from normal parameters, addressing appropriately and timely the modifiable factors can potentially reduce the risk of chronic diseases and slow age-related decline.

Incorporating in clinical practice the easily accessible IIM biomarkers that have a significant ability to predict the occurrence and progression of different classes of disorders before they present clinically could provide clinicians with auxiliary data for the early prognosis/diagnosis of musculoskeletal and related diseases, the risks of falls and fractures, and allow for the timely consideration/introduction of preventive measures, particularly in older adults prone to multimorbidity. Therefore, we recommend adding these simple and useful tests to the routine arsenal of diagnostic modalities when evaluating patients for OP/OF risk, identifying which particular IIM component(s) is abnormal and may be predisposed to chronic diseases, including OP/OF; this appears especially important in individuals with suspected musculoskeletal fragility risk.

The advantage of such an approach, which is quite different from the traditional screening for OP/OF risk, is supported by the following lines of scientific clinical evidence.

(1) Data on mechanistic link between deregulated IIM homeostasis and OP/OF in the general population and individuals with various chronic diseases: lower BMD/OP is associated with lower haemoglobin levels [322,323], high neutrophils [322,324], low lymphocytes [322,325,326], low platelets [327], macrophage/monocyte dysfunction [18,328,329,330], elevated RDW levels [331,332,333,334,335,336,337,338,339,340,341], NLR and PLR [234,342,343,344,345,346], LMR [234], SII and SIRI [103,347], serum cytokines [348,349,350,351], metabolomic changes, including elevated GGT [22,27,352,353,354], hypoalbuminaemia [355], disbalanced adipokines, vitamin and mineral deficiencies, oxidative stress, and other indices of IIM dysregulation [26,356,357,358,359,360,361,362,363]. In other words, indices of IIM deregulation are associated with increased likelihood of developing OP/OF and, importantly, many of these factors are potentially reversible or modifiable (potential therapeutic targets) and should be routinely assessed and managed; initiating appropriate preventive measures may simultaneously reduce the risks of OP/OF and numerous related chronic diseases.
(2) Many chronic diseases are bi/multi-directionally linked to the development and progression of musculoskeletal loss, falls, and fractures, and they also contribute to outcomes, displaying a vicious cycle between musculoskeletal status and chronic disorders. Indeed, CVD [364,365,366,367,368,369,370,371,372], CKD [373,374,375,376,377], T2DM [378], CLD [379,380,381,382,383,384], neurodegenerative diseases [385,386,387,388,389,390], COPD [391,392,393,394], gut dysbiosis [395,396,397,398,399,400], and cancer [401,402,403], (to name a few) are associated with decreased physical functioning, frailty, OP, injurious falls, and OFs, whereas impaired osteogenesis (i.e., decline of osteoblasts), altered production of osteokynes (i.e.,osteocalcin, osteoprotegerin, osteopontin) and myokines affect all vital functions of the organism, including haemopoiesis in the bone marrow (reduction in both lymphoid and myeloid cells) [322,345], endocrine, liver, renal, muscles, other functions [404,405,406,407,408,409,410,411,412,413,414,415,416], and OP/OFs; alterations in metabolism affect the immune system and vice versa [10,15,20,417,418,419,420,421,422,423,424]. OP/OFs in turn increase the risk of and affect the progression of chronic disease, decrease quality of life and lifespan, accelerate mortality risk, and increase health care costs.
(3) Impaired IIM homeostasis is a common but often overlooked determinant of numerous chronic disorders (commonly asymptomatic in the early stages) which are linked to musculoskeletal deterioration, falls and fractures, accelerated biological ageing (“inflammageing”), declined resilience, and frailty. The risk of the onset and progression of the above-mentioned disorders can be predicted using IIM indices. From a clinical and pathophysiological point of view, particularly useful elements might include haemoglobin (Hb), complete blood cell count [148,157,166,167,170,177,178,179,181,183,188,274,425,426,427], RDW [331,332,334,335,336,338,428,429,430], and indexes of systemic inflammation, such as NLR [330,430,431], PLR [268], LMR [330], SII [282,297,432,433], SIRI [330] (alone or integrated in an inflammatory prognostic scoring system [434]), and hypoalbuminaemia [208,223,435,436,437]; moreover, most abnormal single and combined IIM markers are associated with low vitamin D levels [438], a pluripotent hormone involved in the pathophysiology of OP/OF and multiple chronic diseases [439,440,441].

The importance and potential benefits of clinical investigation of IIM status and treating changes (e.g., low-grade inflammation) as a preventive strategy should be emphasised [4,442,443,444]. In the US population, for instance, the proportion of adults with systemic inflammation is 34.63%; the proportion of individuals aged ≥20 years with no disease is 15.1%, with undiagnosed disease—29.1%, and with diagnosed disease and inflammation —41.8% [444]. The pro-inflammatory response, known as an evolutionary advantage (eradication of infections, promotion wound healing, etc.), especially in times of nutritional deficiency, in the current human society often contributes to metabolic disorders (osteoporosis, obesity, insulin resistance, atherosclerosis, etc.) and their complications. Subtle/small but notable changes in IIM status appear as valuable markers in assessing the likelihood of the onset of chronic disorders but are undervalued and/or under-reported.

Despite ongoing efforts to reduce the OP/OF burden, currently, early identification of conditions that can predispose to OP/OF and accurate estimation of fracture risk, particularly in older adults, remain challenging, and population screening is still a matter of debate [445,446,447,448,449,450,451,452,453,454].

In the general population (including patients with pathologies associated secondarily with OP), more than half of fractures occur in patients without OP as defined by the BMD T score criteria, currently the “gold standard” for diagnosing OP and assessing fracture risk [376,455,456,457,458,459,460,461,462,463]. Screening for OP, even among aged women, remains low [464,465]; prior to fracture, approximately 70–85% of patients with HF and 98% with vertebral fracture had not been diagnosed with OP, and only 16.4% [466] and 21% [467] had been on OP treatment, respectively. Untreated OP leads to a vicious cycle of recurrent fracture(s) [468].

Over six decades of age, risk of HF rises 100- to 1000-fold, but only a minor part of this increase is explained by declining BMD [469]. A recent systematic review on current risk prediction tools for primary prevention of fragility fractures among adults aged ≥40 years concluded that BMD measurement and FRAX risk assessment are of limited benefit; even screenings of postmenopausal females resulted in only a small reduction in OFs [451]. Treatment based only on osteoporotic BMD could not reduce the large number of fractures in the general population [470]. Moreover, how and when to initiate treatment is still a controversial issue, and the effects of current preventive strategies/therapies are suboptimal [470,471,472].

Heightened IIM dysregulation(s) may affect directly and indirectly multiple organ systems and play a critical role in the musculoskeletal remodelling by starting and maintaining the pathological cascade, which may result in osteosarcopenia [473,474,475,476,477,478,479] and correlate with adverse outcomes/mortality risk. Individuals with abnormal/aberrant (albeit silent) IIM parameters should be aware of the potential risks of chronic disorders, including OP/OF (even in the absence of standard diagnostic criteria for OP). The IIM status should be regularly monitored to identify those at risk of disease in a timely manner, and therapeutic attempts should be made to target abnormalities. The IIM biomarkers may contribute to the progress in precision medicine in complex and heterogeneous diseases.

IIM dysregulation(s), which can be detected before and without BMD evidence of OP and/or FRAX assessment, exhibit desirable curative prospects. In a substantial portion of patients, IIM biomarkers could provide more detailed information on altered pathways and suggest more holistic and personalised targeted management, simultaneously influencing the modifiable risk factors for multiple disorders, including OP/OFs.

The practicality and informativeness of IIM haematological indices and a number of directions along which this line of work can be extended have been briefly discussed above. The presence of IIM dysregulation may require multidisciplinary care due to susceptibility to various health issues (various comorbidities). Initiating appropriate preventive measures by addressing specific modifiable causes of these abnormalities (e.g., vitamin D and/or K deficiency, malnutrition, hypoalbuminaemia, anaemia, unhealthy lifestyle factors, decreased mobility, low-grade inflammation (i.e., caused by periodontitis), treating chronic extra-skeletal disorders, etc.) may have a fundamental (direct and indirect) effect on preventing OP/OFs.

Identification and correction of IIM dysregulation(s) may reduce the onset and progression of multiple disorders, including those which are directly and indirectly associated with OP/OFs. However, it remains unclear whether this approach will cause overestimation/over-prediction(s) or underestimation, will result in a positive effect on morbidity and mortality, and will be cost-effective. To prevent unnecessary or incorrect treatments, the decision-making should be evidence-based (avoid inaccurate interpretation of the tests) and individualised. While IIM deregulation can be used as an umbrella term for the spectrum of OP/OF associated disorders, each patient is immunologically and metabolically different; although the components of IIMR are interconnected, their relative impacts on outcome in individual patients vary; correct characterisation and quantifying/grading selected IIM indices in combination with clinical characteristics is therefore important to personalising risk and treatment.

The IIM biomarkers presented can be easily integrated in design and construction of future digital predicting models, which may provide interventions that will further improve multidisciplinary individualised patient management [480,481,482,483].

Further research is needed to deepen, clarify, and validate the conceptual framework of IIM dysregulations as key factors underlying different aspects of the occurrence and development of OP/OF and other orthopaedic and associated diseases.

### 4.3. Strengths and Limitations

The main strengths of this study are as follows: being the first to evaluate and compare (head-to-head) the preoperative prognostic value of 27 blood parameters of IIM homeostasis in a relatively large number of patients with osteoporotic HF; the detailed clinical and laboratory information; and the adjustment for potential/relevant confounders affecting outcomes (including numerous sociodemographic, clinical, and laboratory parameters) in the multivariate models.

The study has also several important limitations. First, the study was conducted in a single tertiary hospital; therefore, it was observational and cannot infer causality. Second, we focused on the prognostic value of on-admission parameters mainly for prediction of postoperative myocardial injury and all-cause in-hospital mortality, and the analysis did not cover other adverse effects; in our cohort there was only 4.8% fatal outcomes, which limited the power of our analyses. Using dynamic (postoperative) IIM changes may further improve the global clinical judgement of patients’ outcomes. Third, the reported cut-offs in the literature range widely, and some cut-offs used in this study were arbitrary; therefore, more research is required to investigate their clinical relevance. Furthermore, the presented predictive models were found to be broadly applicable and need external validation. Finally, the study population was mainly represented by patients of European ancestry, so results may not be generalizable to other racial and ethnic groups.

## 5. Conclusions

The study evaluated and compared the prognostic value of 27 indices of IIM derangement in patients with HF at the time of admission and identified several simple, widely available, and inexpensive parameters with high predictive performance for PMI and in-hospital death; these results/data may help to optimise clinical decisions (i.e., distinguish between patients who may benefit from surgical treatment and those who will not) and provide an individualised therapeutic approach.

Evolving evidence suggests that assessing IIM status in the general population in primary care settings (before an individual become clinically symptomatic) might present suitable biomarkers to predict, diagnose, and manage risks and outcomes for a whole range of chronic diseases, including OP/OF, in the preclinical stages.

## Figures and Tables

**Table 1:** Selected immuno-inflammatory–metabolic indices and used cut-offs.

**Single Parameters (Absolute Values)**	
RBC	<4.30 × 10^12^/L (lower limit of reference range)
Anaemia	haemoglobin <130 g/L (men) and <120 g/L (women)
Neutrophils	>7.5 × 10^9^/L (upper limit of reference range)
Lymphocytes	<1.1 × 10^9^/L (lower limit of reference range)
Monocytes	>1.0 × 10^9^/L (upper limit of reference range)
Platelets	>400 × 10^9^/L (upper limit of reference range)
Eosinophils	<0.5 × 10^9^/L (median)
Red cell distribution width (RDW)	>14.5% (upper limit of reference range)
Albumin	<33 g/L (lower level of reference range)
**Composite parameters (ratios or products)**	
NLR	>7.5 (median)
PLR	>280.0 (4th quartile),
LMR	<1.1 (1st quartile)
SII	1620.0 (median)
SIRI	>5.1 (median)
Mon/Eos ratio	>13.0 (median)
Neutr/Eos ratio	>156.3 (median)
Neutr/Mon ratio	>12.1 (median)
Neutr/Alb × 10	>2.4 (median)
Alb/RDW ratio	<2.6 (median),
Hb/RDW ratio	<8.8 (median)
RDW/Plt × 100	>6.6 (median)
Hb/Alb ratio	>4.6 (median)
Alb × Lymph	<25.4 (median)
ALT/Lymph ratio	<14.6 (median)
GGT/Lymph ratio	>25.4 (median)
Plt/Alb ratio	>5.9 (median)
Plt/ALT ratio	>13.8 (median)

*Abbreviations:* Eos, eosinophil; Lymph, lymphocyte; Mon, monocyte; Neutr, neutrophil; Plt, platelet; Hb, haemoglobin, RDW, red cell distribution width; Alb, albumin; ALT, alanine aminotransferase; Alb/RDW, albumin/red blood cell distribution width ratio; ALT/Lymph, alanine aminotransferase/lymphocyte ratio; Alb × Lymph, albumin × lymphocyte multiplication; GGT, gamma-glutamyl transferase; GGT/Lymp, gamma glutamyl transferase/lymphocyte ratio; Hb/RDW, haemoglobin/red blood cell distribution width ratio; Hb/Alb, haemoglobin/albumin ratio; LMR, lymphocyte/monocyte ratio; Mon/Eos, monocyte/eosinophil ratio; Neutr/Alb, neutrophil/albumin ratio; NLR, neutrophil/lymphocyte ratio; Neutr/Eos, neutrophil/eosinophil ratio; Neutr/Mon, neutrophil/ monocyte ratio; PLR, platelet/lymphocyte ratio; PLT/ALT, platelet/alanine aminotransferase ratio; Plt/Alb, platelet/albumin ratio; RDW/Plt × 100, red blood cell distribution width/platelet ratio (multiplied by 100); SII, systemic immune–inflammation index; SIRI, system inflammation response index. The units of neutrophils, lymphocytes, monocytes, eosinophils, and platelets were all 10^9^/L; ALT and GGT are expressed in IU. The ratios were calculated by dividing the numerator by the denominator (the values for each were expressed in units as shown). Formulas used for calculating integrated indices: SII = platelet count × neutrophil count/lymphocyte count, SIRI = neutrophil count × monocyte count /lymphocyte count. To simplify, all cut-offs were rounded to the nearest tenth.

**Table 2:** Comparison of demographic and haematological characteristics at admission in hip fracture patients with and without ischaemic heart disease (IHD).

Variable	Total Cohort (n = 1273)	With IHD (n = 361, 28.4%)	Without IHD (n = 912, 71.6%)	*p* Value
Age, mean ± SD, years	82.9 ± 8.7	84.9 ± 7.2	82.2 ± 9.1	**<0.001**
Aged > 80 years, %	70.6	76.7	68.2	**0.001**
Female, %	73.5	69.5	75.0	**0.028**
RBC < 4.30 × 10^12^, %	69.3	72.9	67.9	**0.047**
Anaemia	41.8	44.3	40.8	0.138
Neutr > 7.5 × 10^9^, %	60.1	57.6	61.1	0.142
Lymp < 1.2 × 10^9^, %	58.4	58.5	58.5	0.525
Eos < 0.5 × 10^9^, %	53.9	61.5	50.8	**<0.001**
Mon > 1.0 × 10^9^, %	15.9	19.1	14.6	**0.046**
Plt > 400 × 10^9^, %	3.2	2.3	3.6	0.156
RDW > 14.5%, %	37.5	49.6	32.7	**<0.001**
Albumin < 33 g/L, %	19.7	18.9	20.0	0.358
NLR > 7.5, %	50.1	49.9	50.2	0.479
PLR > 280, %	25.9	25.3	26.1	0.41
LMR < 1.1, %	25.2	28.3	24	0.068
SII > 1620, %	50.0	48.0	50.8	0.208
SIRI > 5.1, %	50.0	51.3	49.5	0.303
Hb/RDW < 8.8, %	50.0	57.3	47.2	**0.001**
RDW/Plt × 100 > 6.6, %	50.0	57.9	46.9	**0.001**
Alb/RDW < 2.6, %	50.0	55.8	47.7	**0.005**
Neutr/Eos > 156.3, %	50.0	57.1	47.2	**0.001**
Neutr/Mon > 12.4, %	50.0	47.4	51	0.135
Mon/Eos > 13.0, %	50.1	57.1	47.1	**0.001**
Neutr/Alb × 10 > 2.4, %	50.0	50.0	50.0	0.525
Hb/Alb > 4.6, %	49.8	47.8	50.6	0.204
Plt/Alb > 5.9, %	49.2	45.4	50.8	**0.048**
Plt/ALT > 13.8, %	50.0	48.5	50.6	0.271
Alb/Lymp < 25.4,%	50.0	49.7	50.1	0.475
ALT/Lymp < 14.6, %	50.0	52.5	49.0	0.475
GGT/Lymp > 25.4,%	49.9	52.8	48.8	0.112

*Abbreviations*: IHD, ischaemic heart disease; anaemia, Hb < 120 g/L in females, <130 g/L in males; all other abbreviations as in Table 1. Significant *p* values are expressed in bold characters.

**Table 3:** Haematological characteristics at admission and hospital outcomes (postoperative myocardial injury or death) in hip fracture patients.

Variable	Total Cohort (n = 1273)	Postoperative Myocardial Injury		*p* Value	Survivors (n = 1212, 95.2%)	Died (n = 61, 4.8%)	*p* Value
		Yes (n = 555, 43.6%)	No (n = 912, 71.6%)				
Age, mean ± SD, years	82.9 ± 8.7	86.1 ± 6.8	80.8 ± 8.9	**<0.001**	82.7 ± 8.7	88.1 ± 6.1	**<0.001**
Aged > 80 years, %	70.6	85.2	60.4	**<0.001**	69.6	91.8	**<0.001**
Female, %	73.5	42.1	57.8	0.054	73.7	68.9	0.243
RBC < 4.30 × 10^12^/L, %	69.3	70.3	68.8	0.306	69.4	67.2	0.408
Anaemia (Hb < 120/130 g/L), %	41.8	46.0	38.4	**<0.001**	41.2	54.1	**0.032**
Neutr > 7.5 × 10^9^, %	60.1	62.5	58.8	0.109	59.7	67.2	0.151
Lymp < 1.2 × 10^9^, %	58.4	62.9	55.5	**0.006**	58.2	63.9	0.225
Eos < 0.5 × 10^9^, %	53.9	47.7	44.7	0.165	45.3	62.3	**0.007**
Mon > 1.0 × 10^9^, %	15.9	18.7	13.6	**0.009**	15.9	14.8	0.489
Plt > 400 × 10^9^, %	3.2	2.7	3.5	0.247	3.1	5.0	0.298
RDW > 14.5%, %	37.5	43.0	32.5	**<0.001**	36.5	57.4	**0.001**
Albumin < 33 g/L, %	19.7	18.1	20.1	0.198	19.5	23.0	0.303
NLR > 7.5, %	50.1	55.7	46.7	**0.001**	49.4	63.9	**0.018**
PLR > 280, %	25.9	30.2	22.5	**0.002**	25.0	43.3	**0.002**
LMR < 1.1, %	25.2	31.5	20.6	**<0.001**	24.3	42.6	**0.002**
SII > 1620.0, %	50	54.9	46.3	**0.002**	49	70.0	**0.001**
SIRI > 5.1, %	50	56.3	45.8	**<0.001**	49.1	67.2	**0.004**
Hb/RDW < 8.8, %	50	55.4	45.6	**0.001**	49	70.5	**0.001**
RDW/Plt × 100 > 6.6, %	50	53.0	48.4	0.061	49.8	53.3	0.346
Alb/RDW < 2.6, %	50	53.6	45.2	**0.002**	49.9	72.1	**<0.001**
Neutr/Eos > 156.3, %	50.1	51.4	48.4	0.302	49	70.5	**0.001**
Neutr/Mon > 12.4, %	50	50.8	50.6	0.486	49.4	60.7	0.057
Mon/Eos > 13.0, %	50.1	51.2	48.9	0.229	49.5	62.3	**0.034**
Neutr/Alb × 10 > 2.4, %	50	53.4	46.8	**0.013**	49.4	62.3	**0.033**
Hb/Alb > 4.6, %	49.8	47.9	52	0.087	50.5	36.1	**0.019**
Plt/Alb > 5.9, %	49.2	47.2	49.4	0.245	48.5	63.3	**0.017**
Plt/ALT > 13.8, %	50	51.2	49	0.235	49.7	55.0	0.252
Alb/Lymp < 25.4,%	50	53.0	48.1	0.09	49.5	60.7	0.057
ALT/Lymp < 14.6, %	50	51.2	48.3	0.176	49.5	60.7	0.088
GGT/Lymp >25.4, %	49.9	49.3	50.3	0.381	49.3	62.3	**0.032**

*Abbreviations*: as in Table 1. Significant *p* values are expressed in bold characters.

**Table 4:** Clinical profile and laboratory characteristics at admission in hip fracture patients who developed PMI: comparison IHD and non-IHD groups.

Clinical Variables	Total Cohort (n = 533)	IHD		*p* Value	Laboratory Variables	IHD		*p* Value
		Yes	No			Yes	No	
Age, mean ± SD, years	86.14 ± 6.8	86.7 ± 6.52	85.8 ± 6.99	0.157	RBC < 4.30 × 10^12^, %	75.1	67.5	0.061
Aged > 80 years, %	83.5	83.1	83.7	0.845	Anaemia, %	49.3	44.0	0.236
Female, %	71.1	66.2	74.1	**0.050**	Neutr > 7.5 × 10^9^, %	59.7	64.2	0.303
PRCF resident, %	38.7	39.3	38.4	0.830	Lymp < 1.2 × 10^9^, %	61.2	63.9	0.538
HF type [trochanteric], %	48.2	49.8	47.3	0.581	Eos < 0.5 × 10^9^, %	61.7	46.7	**0.001**
History of AMI, %	9.3	23.4	NA	NA	Mon > 1.0 × 10^9^, %	19.9	18.1	0.600
Hypertension, %	60.2	64.7	57.5	0.102	Plt > 400 × 10^9^, %	1.5	3.4	0.204
CVA, %	11.8	12.9	11.4	0.535	RDW > 14.5%, %	49.8	38.9	**0.014**
TIA, %	12.6	11.9	13.0	0.733	Albumin < 33 g/L, %	16.4	19.0	0.447
CKD, %	44.3	54.7	37.9	**<0.001**	NLR > 7.5, %	54.2	56.6	0.589
COPD, %	16.5	20.9	13.9	**0.034**	PLR > 280, %	28.8	31.1	0.576
Anaemia, %	46.0	49.3	44.0	0.236	LMR < 1.1, %	31.8	31.3	0.901
T2DM, %	24.1	28.0	21.3	0.068	SII > 1620.0, %	51.5	57.0	0.220
Dementia, %	38.5	33.3	41.6	0.056	SIRI > 5.1, %	54.2	57.5	0.456
Parkinson’s disease, %	3.8	2.5	4.5	0.232	Hb/RDW < 8.8,%	60.7	52.1	**0.053**
Smoker, %	4.1	3.5	4.5	0.560	RDW/Plt × 100 > 6.6, %	59.1	49.4	**0.031**
Ex-smoker, %	13.0	10.5	14.5	0.177	Alb/RDW < 2.6, %	55.7	52.3	0.438
* Alcohol over-user, %	1.9	1.0	2.4	0.243	Neutr/Eos > 156.3, %	58.2	42.5	**<0.001**
Walking aids user, %	38.7	40.3	37.7	0.543	Neutr/Mon > 12.1, %	47.3	53.0	0.198
In-hospital mortality, %	8.8	11.0	7.5	0.178	Mon/Eos > 13.0, %	57.8	40.3	**<0.001**
Postoperative AMI, %	15.4	19.9	12.7	**0.025**	Neutr/Alb × 10 > 2.4, %	53.2	53.5	0.957
LOS > 10 days, %	61.4	59.2	62.7	0.428	Hb/Alb > 4.6, %	46.8	48.6	0.675
LOS > 20 days, %	22.1	24.9	20.5	0.236	Plt/Alb > 5.9, %	41.9	50.5	0.057
CRP > 100 mg/L, %	88	87.5	88.3	0.796	Plt/ALT > 13.8, %	49.5	52.3	0.534
CRP > 150 mg/L, %	69.2	67.0	70.5	0.400	Alb/Lymp < 25.4,%	49.7	45.3	0.32
					ALT/Lymp < 14.6, %	50.3	47.1	0.485
					GGT/Lymp > 25.4, %	54.2	46.2	0.073

*Abbreviations:* For clinical characteristics and outcomes: PRCF, permanent residential care facility; IHD, ischaemic heart disease; AMI, acute myocardial infarction; CKD, chronic kidney disease (estimated glomerular filtration rate < 60 mL/min/1.73 m^2^); CVA, cerebrovascular accident (stroke); TIA, transient ischaemic attack; COPD, chronic obstructive airway disease; T2DM, type 2 diabetes mellitus; LOS, length of hospital stay; CRP, c-reactive protein; *, alcohol consumption ≥ 3 times per week; for haematological characteristics, all abbreviations as in Table 1. Significant *p* values are expressed in bold characters.

**Table 5:** Prognostic value of age, presence of IHD, and specific laboratory characteristics at admission for predicting postoperative outcome in patients with hip fracture.

**Postoperative Myocardial Injury**						
**Variable**	**^1^ Total Cohort (n = 1273)**		**^2^ IHD (n = 361)**		**^3^ IHD > 80 Years of Age (n = 277)**	
	**OR (95% CI)**	***p* Value**	**OR (95% CI)**	***p* Value**	**OR (95% CI)**	***p* Value**
Age > 80 years	3.84 (2.83–4.99)	**<0.001**	3.95 (2.92–5.27)	<0.001	NA	NA
IHD	2.30 (1.81–3.01)	**<0.001**	NA	NA	8.3 (5.58–12.36)	**<0.001**
LMR < 1.1	1.56 (1.18–2.06)	**0.002**	3.84 (2.44–6.05)	**<0.001**	16.08 (8.54–30.29)	**<0.001**
PLR > 280.0	1.44 (1.09–1.90)	**0.01**	3.87 (2.38–6.29)	**<0.001**	16.36 (8.44–11.74)	**<0.001**
Anaemia	1.08 (0.84–1.38)	0.568	3.37 (2.31–4.93)	**<0.001**	14.13 (8.06–24.78)	**<0.001**
Mon/Eos > 13.0	1.30 (1.01–1.66)	**0.038**	2.50 (1.70–3.68)	**<0.001**	13.43 (6.81–26.49)	**<0.001**
NLR > 7.5	1.40 (1.10–1.79)	**0.006**	3.47 (2.40–5.02)	**<0.001**	12.42 (7.03–21.94)	**<0.001**
Eos < 0.5 × 10^9^/L	1.38 (1.08–1.77)	**0.01**	2.77 (1.85–4.14)	**<0.001**	12.29 (6.44–23.47)	**<0.001**
SIRI > 5.1	1.42 (1.11–1.81)	**0.005**	3.44 (2.40–5.00)	**<0.001**	11.99 (6.81–21.09)	**<0.001**
Neutr/Alb × 10 > 2.4	1.25 (0.98–1.60)	0.068	3.22 (2.22–4.65)	**<0.001**	11.74 (6.60–20.88)	**<0.001**
Lymp < 1.2 × 10^9^/L	1.26 (0.99–1.62)	0.065	3.14 (2.20–4.48)	**<0.001**	11.69 (6.57–20.79)	**<0.001**
Monocytes > 1 × 10^9^/L	1.33 (0.96–1.85)	0.089	2.93 (1.73–5.00)	**<0.001**	11.61 (5.72–23.56)	**<0.001**
GGT/Lymp > 25.4	0.95 (0.74–1.21)	0.658	2.32 (1.63–3.30)	**<0.001**	11.17 (6.23–20.05)	**<0.001**
SII > 1650	1.41 (1.10–1.801)	**0.006**	3.33 (2.41–5.16)	**<0.001**	10.51 (5.96–18.53)	**<0.001**
Alb/RDW < 2.6	1.09(0.85–1.39)	0.493	2.94 (2.07–4.17)	**<0.001**	10.20 (6.01–17.33)	**<0.001**
RDW/Plt × 100 > 6.6	1.06 (0.83–1.36)	0.642	2.50 (1.78–3.51	**<0.001**	9.93 (5.74–17.17)	**<0.001**
Plt/ALT > 13.8	0.96 (0.75–1.23)	0.750	2.69 (1.85–3.91)	**<0.001**	9.87 (5.65–12.27)	**<0.001**
Neutr > 7.5 × 10^9^/L	1.21 (0.94–1.55)	0.139	2.82 (1.97-4.05)	**<0.001**	9.61 (5.26–17.58)	**<0.001**
RDW > 14.5%	1.17 (0.91–1.50)	0.227	2.84 (2.00–4.04)	**<0.001**	9.51 (5.73–15.79)	**<0.001**
Neutr/Mon > 12.1	1.10 (0.86–1.41)	0.434	2.37 (1.64–3.42)	**<0.001**	8.49 (4.74–15.21)	**<0.001**
ALT/Lymph < 14.6	1.28 (1.01–1.64)	**0.045**	2.06 (1.45–2.93)	**<0.001**	7.23 (4.21–12.40)	**<0.001**
Alb < 33 g/L	0.80 (0.59–1.09)	0.162	1.80 (1.07–3.02)	**0.026**	6.64 (3.37–13.08)	**<0.001**
Alb/Lymph/ < 25.4	0.96 (0.75–1.22)	0.728	2.10 (1.46–3.01)	**<0.001**	6.56 (3.64–11.85)	**<0.001**
Plt/Alb ratio > 5.9	0.89 (0.70–1.1)	0.351	2.12 (1.45–3.10)	**<0.001**	6.07 (3.50–10.53)	**<0.001**
Neutr/Eos > 156.3	1.38 (1.08–1.77)	**0.01**	2.05 (1.46–2.87)	**<0.001**	5.34 (3.27–8.74)	**<0.001**
Hb/Alb > 4.6	1.07 (0.84–1.37)	0.593	2.00 (1.39–2.88)	**<0.001**	6.03(3.34–10.89)	**<0.001**
Platelets > 400 × 10^9^/L	0.92 (0.46–1.89)	0.835	0.99 (0.23–4.15)	0.985	3.38 (0.73–15.62)	0.099
Hb/RDW < 8.8	0.91 (0.71–1.17)	0.465	1.64 (1.12–2.39)	**0.011**	4.98 (2.60–9.51)	**<0.001**
**In-Hospital Death**						
**Variable**	**^1^ Total Cohort (n = 1273)**		**^2^ IHD (n = 361)**		**^3^ IHD > 80 Years of Age (n = 277)**	
	**OR (95% CI)**	***p* Value**	**OR (95% CI)**	***p* Value**	**OR (95% CI)**	***p* Value**
Age > 80 years	4.90 (1.95–12.33)	**0.001**	5.04 (1.96–12.62)	**<0.001**	NA	NA
IHD	2.10 (1.24–3.51)	**0.005**	NA	NA	7.4 (2.55–21.51)	**<0.001**
LMR < 1.1	2.03 (1.18–3.49)	**0.010**	5.19 (2.65–10.15)	**<0.001**	28.67 (6.39–128.70)	**<0.001**
PLR > 280.0	2.16 (1.26–3.68)	**0.005**	5.58 (2.79–11.16)	**<0.001**	29.21 (6.49–131.42)	**<0.001**
Anaemia	1.35 (0.79–2.30)	0.270	3.39 (1.64–7.02)	**0.001**	13.24 (2.97–58.98)	**0.001**
Mon/Eos > 13.0	2.02 (1.17–3.48)	**0.011**	3.93 (1.86–8.30)	**<0.001**	10.99 (2.48–48.69)	**<0.001**
NLR > 7.5	1.72 (1.00–2.96)	**0.051**	3.62 (1.84–7.10)	**<0.001**	5.50 (2.01–15.07)	**<0.001**
Eos < 0.5 × 10^9^/L	2.44 (1.42–4.21)	**0.001**	4.76 (2.30–9.84)	**<0.001**	13.96 (3.16–61.61)	**0.001**
SIRI > 5.1	2.13 (1.23–3.67)	**0.007**	3.95 (2.03–7.66)	**<0.001**	6.18 (2.27–16.77)	**<0.001**
Neutr/Alb × 10 > 2.4	1.69 (1.00–2.88)	**0.051**	3.46 (1.72–6.98)	**0.001**	23.06(3.04–175.15)	**0.002**
Lymp < 1.2 × 10^9^/L	1.18 (0.68–2.03)	0.561	2.54 (1.27–5.08)	**0.008**	18.6 (2.46–140.86)	**0.005**
Monocytes > 1.0 × 10^9^/L	0.91 (0.44–1.89)	0.807	1.22 (0.36–4.12)	0.75	3.35 (0.54–20.56)	0.192
GGT/Lymp > 25.4	1.70 (1.00–2.89)	**0.050**	3.34 (1.66–6.72)	**0.001**	20.53 (2.70–156.06)	**0.004**
SII > 1650.0	2.33 (1.32–4.13)	**0.004**	4.63 (2.26–9.48)	**<0.001**	5.33 (1.95–14.63)	**<0.001**
Alb/RDW < 2.6	2.71 (1.53–4.79)	**0.001**	2.23 (1.25–3.98)	**0.007**	5.42 (2.41–12.19)	**<0.001**
RDW/Plt × 100 > 6.6	1.04 (0.61–1.78)	0.882	2.13 (1.04–4.35)	**0.038**	5.23 (1.47–18.57)	**0.005**
Plt/ALT > 13.8	1.24 (0.73–2.08)	0.424	2.61 (1.34–5.11)	**0.005**	4.87 (1.75–13.57)	**0.001**
Neutrophils > 7.5 × 10^9^/L	1.39 (0.80–2.43)	0.240	2.80 (1.36–5.75)	**0.005**	14.83 (1.96–112.48)	**0.009**
RDW > 14.5%	1.86 (1.09–3.16)	**0.022**	3.93 (1.98–7.79)	**<0.001**	12.10 (3.64–70.42)	**<0.001**
Neut/Mon > 12.1	1.68 (0.98–2.87)	0.057	3.39 (1.67–6.89)	**0.001**	9.94 (2.26–43.72)	**0.002**
ALT/Lymph < 14.6	0.57 (0.33–0.97)	**0.037**	1.30 (0.61–2.76)	0.488	5.75 (1.24–26.69)	**0.026**
Alb < 33 g/L	1.23 (0.66–2.27)	0.512	3.33 (1.45–7.64)	**0.004**	15.17 (3.87–59.53)	**<0.001**
Alb × Lymph/ < 25.4	1.38 (0.81–2.38)	0.233	1.05 (0.43–2.35)	0.921	3.03 (0.62–14.88)	0.173
Plt/Alb ratio > 5.9	1.82 (1.05–3.16)	**0.032**	3.95 (1.83–8.55)	**<0.001**	4.36 (1.55–12.27)	**0.003**
Neutr/Eos > 156.3	2.49 (2.42–4.37)	**0.001**	3.13 (1.75–5.57)	**<0.001**	2.39 (0.61–9.43)	0.199
Hb/Alb > 4.6	1.81 (1.06–3.09)	**0.030**	1.13 (0.40–2.56)	0.768	3.63 (0.75–17.47)	0.108
Platelets > 400 × 10^9^/L	1.65 (0.49–3.51)	0.417	8.43 (1.64–43.38)	**0.011**	35.87 (4.87–263.95)	**<0.001**
Hb/RDW < 8.8	1.92 (1.08–3.40)	**0.025**	1.33 (0.53–3.34)	0.544	2.34 (0.46–11.88)	0.292

*Abbreviations:* As in Table 1, OR, odds ratio; CI, confidence interval; ^1^ adjusted for age, gender and all clinical variables which were significantly associated with postoperative myocardial injury and/or hospital mortality on univariate analyses; ^2^ comparison IHD patients with the rest of the cohort, adjusted for age (as a continues variable) and all clinical variables which were significantly associated with postoperative myocardial injury and/or hospital mortality on univariate analyses; ^3^ comparison IHD patients aged > 80 years and younger than 80 years with and without the analysed haematological characteristic. Significant *p* values are expressed in bold characters.

**Table 6:** Summary of performance parameters of haematological biomarkers at admission to predict postoperative myocardial injury and/or in-hospital mortality in aged (>80 years) hip fracture patients with IHD.

Postoperative Myocardial Injury									
Biomarker	AUC (95% CI)	Sensitivity (%)	Specificity (%)	Accuracy (%)	PPV (%)	NPV (%)	LR+	LR−	NNP
LMR < 1.1	0.7625 (0.7086–0.8163)	61.5	91.0	82.1	74.7	84.5	6.802	0.423	1.69
PLR > 280.0	0.7604 (0.7043–0.8165)	60.7	91.4	82.2	75.0	84.5	7.036	0.430	1.68
Anaemia	0.7879 (0.7400–0.8358)	75.4	82.1	79.7	71.1	85.2	4.225	0.299	1.78
Mon/Eos > 13.0	0.7814 (0.7274–0.8354)	83.1	73.2	76.7	63.3	88.6	3.097	0.231	1.93
NLR > 7.5	0.7784 (0.7299–0.8270)	79.7	76.0	77.5	69.6	84.4	3.220	0.268	1.85
Eos < 0.5 × 10^9^/L	0.7780 (0.7223–0.8337)	78.6	77.0	77.6	66.0	86.4	3.420	0.278	1.91
SIRI > 5.1	0.7753 (0.7667–0.8239)	79.5	75.6	77.1	68.4	84.7	3.253	0.271	1.88
Neutr/Alb × 10 > 2.4	0.7732 (0.7236–0.8229)	79.6	75.0	76.9	68.2	84.6	3.286	0.271	1.89
Lymp < 1.2 × 10^9^/L	0.7643 (0.7172–0.8114)	84.3	68.6	75.3	66.5	85.5	2.684	0.230	1.92
Monocytes > 1.0 × 10^9^/L	0.6933 (0.6342–0.7525)	45.3	93.3	80.7	70.8	82.7	6.800	0.586	1.90
GGT/Lymp > 25.4	0.7671 (0.7165–0.8177)	80.9	72.5	76.1	68.9	83.5	2.946	0.264	1.91
SII > 1620.0	0.7638 (0.7130–0.8146)	78.1	74.7	76.1	68.5	82.9	3.085	0.294	1.95
Alb/RDW < 2.6	0.7613 (0.7136–0.8090)	77.5	74.8	75.8	63.7	85.3	3.071	0.301	2.04
RDW/Plt × 100 > 6.6	0.7557 (0.7075–0.8039)	80.5	70.7	74.6	64.7	84.4	2.743	0.276	2.04
Plt/ALT > 13.8	0.7586 (0.7031–0.8098)	76.1	75.6	75.8	65.9	83.6	3.117	0.316	2.02
Neutr > 7.5 × 10^9^/L	0.7380 (0.6873–0.7887)	85.4	62.2	72.9	66.0	83.2	2.261	0.235	2.03
RDW > 14.5%	0.7514 (0.7030–0.79970	69.9	80.4	76.7	66.2	82.9	3.559	0.374	2.04
Neutr/Mon > 12.1	0.7396 (0.6867–0.7924)	80.2	67.7	72.7	62.5	83.6	2.484	0.293	2.17
ALT/Lymph > 14.6	0.7282 (0.6754–0.7810)	75.2	70.4	72.3	63.0	81.0	2.542	0.352	2.27
Alb < 33 g/L	0.6564 (0.5927–0.7201)	40.6	90.7	79.1	56.5	83.6	4.347	0.655	2.50
Alb/Lymph/ < 25.4	0.7145 (0.6566–0.7723)	78.1	64.8	70.9	65.1	77.9	2.219	0.338	2.33
Plt/Alb ratio > 5.9	0.7112 (0.6544–0.7681)	71.0	71.3	71.2	60.7	79.7	2.479	0.407	2.48
Hb/RDW < 8.8	0.6813 (0.6165–0.7461)	78.3	57.9	66.8	59.1	77.5	1.862	0.374	2.73
Hb/Alb < 4.6	0.7058 (0.6469–0.7647)	77.5	63.7	69.9	63.7	77.5	2.134	0.354	2.43
Plt > 400 × 10^9^/L	0.5207 (0.4863–0.5552)	6.0	98.1	80.8	42.9	81.9	3.249	0.959	4.03
Neutr/Eos > 156.3	0.6972 (0.6458–0.7487)	72.6	66.9	69.4	62.8	76.0	2.190	0.410	2.58
**In-Hospital Death**									
LMR < 1.1	0.8375 (0.7553–0.9197)	88.2	79.3	79.7	19.5	99.2	4.255	0.148	5.35
PLR > 280.0	0.8390 (0.7566–0.9214)	88.2	79.6	80.1	20.8	99.1	4.319	0.148	5.03
Anaemia	0.7604 (0.6770–0.8438)	88.2	63.8	65.1	11.9	99.0	2.440	0.184	9.17
Mon/Eos > 13.0	0.7293 (0.6514–0.8072)	89.5	56.4	58.9	14.7	98.5	2.055	0.187	7.58
NLR > 7.5	0.6506 (0.5678–0.7335)	80.0	57.9	59.7	14.3	97.1	1.900	0.345	8.77
Eos < 0.5 × 10^9^/L	0.7540 (0.6794–0.8285)	90.0	60.8	63.2	16.8	98.6	2.296	0.164	6.49
SIRI > 5.1	0.6614 (0.5813–0.7416)	81.5	58.4	60.4	15.9	97.1	1.958	0.317	7.69
Neutr/Alb × 10 > 2.4	0.7545 (0.6952–0.8138)	94.7	56.2	58.6	12.9	99.4	2.161	0.094	8.13
Lymp < 1.2 × 10^9^/L	0.7224 (0.6656–0.7792)	95.0	49.1	52.1	11.4	99.3	1.868	0.102	9.34
Monocytes > 1.0 × 10^9^/L	0.6169 (0.3760–0.8579)	40.0	83.4	82.7	3.9	98.8	2.408	0.720	37.03
GGT/Lymp > 25.4	0.7401 (0.6802–0.8000)	94.7	53.3	56.1	12.9	99.3	2.028	0.099	8.20
SII > 1620.0	0.6795 (0.5947–0.7643)	80.0	57.1	59.1	14.6	96.9	1.867	0.350	8.70
Alb/RDW < 2.6	0.6991 (0.6093–0.7888)	78.3	57.6	59.0	11.6	97.4	1.845	0.378	11.11
RDW/Plt × 100 > 6.6	0.6760 (0.5784–07736)	82.4	52.8	54.4	9.0	98.1	1.746	0.334	14.08
Plt/ALT > 13.8	0.6103 (0.527–0.6939)	77.3	58.9	60.2	12.6	97.1	1.879	0.386	11.49
Neutr > 7.5 × 10^9^	0.6942 (0.6367–0.7518)	95.0	43.8	47.5	11.5	99.1	1.692	0.114	9.26
RDW > 14.5%	0.7739 (0.6987–0.8491)	89.5	65.3	66.6	12.5	99.1	2.579	0.161	8.62
Neutr/Mon > 12.1	0.7125 (0.6385–0.7864)	90.0	52.5	55.2	12.7	98.6	1.894	0.191	8.85
ALT/Lymph > 14.6	0.6840 (0.5702–0.7979)	83.3	53.5	54.7	6.9	98.7	1.791	0.312	17.86
Alb < 33 g/L	0.7889 (0.6493–0.9285)	72.7	85.1	84.6	16.0	98.8	4.866	0.321	6.76
Alb/Lymph/ < 25.4	0.6208 (0.4733–0.7684)	77.8	46.4	47.5	5.3	98.2	1.451	0.479	25.00
Plt/Alb > 5.9	0.6596 (0.5646–0.7546)	76.2	57.7	59.1	12.7	96.8	1.801	0.413	10.53
Hb/RDW < 8.8	0.5941 (0.4299–0.7582)	75.0	43.8	45.0	5.2	97.7	1.335	0.571	34.48
Hb/Alb < 4.6	0.6379 (0.5032–0.7726)	80.0	47.6	48.9	6.3	98.2	1.526	0.420	22.20
Plt > 400 × 10^9^	0.6909 (0.4507–0.9311)	40.0	98.2	97.1	2.9	98.9	21.920	0.611	21.28
Neutr/Eos > 156.3	0.6032 (0.4510–0.7555)	70.0	50.6	51.2	4.4	98.1	1.418	0.592	40.00

*Abbreviations:* AUC, area under the curve (receiver operating characteristic); PPV, positive predictive value; NPV, negative predictive value; LR+, positive likelihood ratio; LR−, negative likelihood ratio; all other abbreviations as in Table 1. Calibration of the models (Hosmer–Lemeshow goodness-of-fit test) was good (0.1900 and above, *p* > 0.100).

**Table 7:** The literature data on the prognostic value of selected blood parameters to predict postoperative mortality in patients with hip fracture (1) and adverse outcomes in patients with IHD/CVDs (2).

Parameter/Index	Hip Fracture (1)	IHD/CVDs (2)
Anaemia	[127,130,131,132,133,134,135,136,137] No: [122]	[138,139] No: [140]
Neutrophils elevated	[141,142]	[143,144,145,146,147,148,149]
Lymphocytes low	[130,131,150,151,152,153,154]	[147,155,156,157] *
Monocytes elevated		[138,147,148,157,158,159,160,161,162,163]
Platelets low	[164] No: [165]	[164,166,167,168] No: [169,170,171,172] *
Eosinophils low	[173]	[155,161,174,175,176,177,178,179,180,181,182,183,184] No: [146,156,157,185,186,187,188,189]
Red cell distribution width (RDW) elevated	[134,190,191,192,193,194,195,196,197,198]	[148,170,197,199,200,201,202,203,204,205]
Albumin low	[131,137,151,153,154,198,206,207,208,209,210,211,212,213,214,215,216,217,218] No: [219]	[220,221,222,223,224,225] No: [226]
NLR elevated	[29,30,227,228,229,230,231,232,233,234,235,236,237,238] No: [137,239,240]	[31,32,149,161,227,238,241,242,243,244,245,246,247,248,249,250,251,252,253,254,255,256,257,258,259,260,261,262,263,264,265] No: [266,267]
PLR elevated	[218,236,268,269] No: [237]	[32,256,260,265,270,271,272] No: [266]
LMR low	[230,234,236,238]	[160,238,258,259,265,273,274,275,276]
SII elevated	[215,238,277,278]	[226,238,260,277,279,280,281,282,283,284]
SIRI elevated		[282,285,286,287]
Mon/Eos ratio elevated		[288,289]
Neutr/Eos ratio elevated		[290,291]
Neut/Alb ratio elevated	[292]	[293,294,295,296,297,298,299,300]
Alb/RDW ratio low		[37,38,301,302,303,304,305]
Hb/RDW ratio low		[306,307]
Alb × Lymph low	[131]	
ALT/Lymph ratio low		[308]
Plt/Alb ratio elevated		[309,310,311]

*Abbreviations:* No, opposite effect reported (e.g., low lymphocyte, or eosinophil counts); *, U-shaped relationship; all other abbreviations as in Table 1.

## Data Availability

The data presented in this study are available upon reasonable request from the corresponding author.
